# The coupled photocycle of phenyl-p-benzoquinone and Light-Harvesting Complex II (LHCII) within the biohybrid system

**DOI:** 10.1038/s41598-022-16892-y

**Published:** 2022-07-27

**Authors:** Magdalena Łazicka, Adriana Palińska-Saadi, Paulina Piotrowska, Bohdan Paterczyk, Radosław Mazur, Magdalena Maj-Żurawska, Maciej Garstka

**Affiliations:** 1 Department of Metabolic Regulation, Faculty of Biology, Institute of Biochemistry, University of Warsaw, Miecznikowa 1, 02-096 Warsaw, Poland; 2 Laboratory of Basics of Analytical Chemistry, Faculty of Chemistry, University of Warsaw, Pasteura 1, 02-093 Warsaw, Poland; 3 Bioanalytical Laboratory, Biological and Chemical Research Centre, University of Warsaw, Żwirki i Wigury 101, 02-089 Warsaw, Poland; 4 Laboratory of Electron and Confocal Microscopy, Faculty of Biology, University of Warsaw, Miecznikowa 1, 02-096 Warsaw, Poland

**Keywords:** Bioenergetics, Antenna complex, Devices for energy harvesting

## Abstract

The combination of trimeric form of the light-harvesting complex II (LHCII_3_), a porous graphite electrode (GE), and the application of phenyl-p-benzoquinone (PPBQ), the quinone derivative, allow the construction of a new type of biohybrid photoactive system. The Chl fluorescence decay and voltammetric analyzes revealed that PPBQ impacts LHCII_3_ proportionally to accessible quenching sites and that PPBQ forms redox complexes with Chl in both ground and excited states. As a result, photocurrent generation is directly dependent on PPBQ-induced quenching of Chl fluorescence. Since PPBQ also undergoes photoactivation, the action of GE-LHCII_3_-PPBQ depends on the mutual coupling of LHCII_3_ and PPBQ photocycles. The GE-LHCII_3_-PPBQ generates a photocurrent of up to 4.5 µA and exhibits considerable stability during operation. The three-dimensional arrangement of graphite scraps in GE builds an active electrode surface and stabilizes LHCII_3_ in its native form in low-density multilayers. The results indicate the future usability of such designed photoactive device.

## Introduction

In plants, light energy is efficiently captured by antenna complexes (LHC) and then transferred to the reaction centers located at the core of photosynthetic complexes, Photosystem II (PSII) and Photosystem I (PSI), where charge separation occurs, and electrons transfer starts^1^. The main peripheral antenna of PSII, the light-harvesting complex II (LHCII), plays a crucial role not only in light harvesting processes, but also in the protection of chlorophyll-protein complexes (CP) by dissipating excess absorbed light energy^2^. LHCII exists in thylakoids in trimeric form (LHCII_3_) wherein every monomer is composed of Lhcb polypeptide, including three transmembrane α-helices, and precisely located in the protein scaffold the molecules of 8 chlorophylls *a* (Chl *a*), 6 chlorophylls *b* (Chl *b*), and 4 xanthophylls^3^.

Some research on artificial photosynthesis concerns biophotovoltaic devices, which are biohybrid systems connecting the CP with the electrodes. In these systems, mainly the PSII, PSI, or bacterial reaction center (BR) are used due to their efficient charge separation and generation of high potential difference between the electron donor and acceptor terminal sites^4–6^. The challenge is to integrate these CP complexes properly with an electrode, enabling unidirectional transfer of electrons and restriction of secondary redox reactions. Reducing the distance between CP and the electrode might be achieved by using a monolayer of complexes placed on a planar electrode surface^7,8^ or application of nanostructured material with a 3-dimensional (3D) porous structure^9,10^.

The electrodes might be built of Au or Ag-based materials or are made of electrically conductive metal oxides such as indium-tin oxide (ITO) or fluorine-doped tin oxide (FTO). If the surfaces of the ITO / FTO electrodes are coated with semiconductors (TiO_2_ or ZnO), the devices containing CP might be qualified as biological dye-sensitized solar cells (DSSC)^6^. Different carbon nanomaterials, among others graphite and graphene, can become the alternative material for the electrode of biohybrid systems due to their high electrical conductivity, broad range of electrochemical potential, and the possibility to design nanostructures with the increased surface area^11–14^.

The challenge, common to all biohybrid systems, is the efficient electron transfer between the complexes and the electrode. One of the solutions is direct electron transfer (DET) by coupling the CP with the electrode by various linking molecules. In mediated electron transfer (MET), diffusing redox reagents are chosen on the basis of their electrochemical potential. Furthermore, various systems combining DET, and MET have been applied^8^.

In thylakoid membranes, the plastoquinone-9 (PQ) molecule consisting of the benzoquinone ring with the polyisoprenoid chain acts as an electron acceptor from PSII^15^. Therefore, the various derivatives of benzoquinone are commonly used as redox mediators undergo reduction by oxidizing the PSII-bound semiquinone^16,17^ or directly from isolated BR^18^. However, these redox properties of quinones are not often utilized in biohybrid systems^19,20^. Independently of their participation in direct redox reactions, quinone derivatives, among others, phenyl-p-benzoquinone (PPBQ), specifically quench the excitation energy in BChl *c* antennas and isolated LHCII^21,22^. However, this property of quinones has not been tested in the performance of biohybrid systems yet.

The LHCII lacks the special Chls pair necessary for charge separation, and the light-harvested energy for further excitation energy transfer is directed to the so-called terminal emitter domain, including a cluster of coupled Chl *a*^23,24^. Thus, the LHCII in vivo is not the source of electron flow. Because of, these complexes have been used as a photosensitizing layer in the construction of DSSC, so far^25–29^. All the results obtained indicate that LHCII is not inferior to other CP complexes used in DSSC construction and can be used to design other biophotovoltaic devices. An additional advantage of LHCII is its smaller size and simpler structure comparing to those of PSI and PSII complexes, which increases its stability and facilitates the introduction of structural modifications.

We have previously found that the generation of photocurrent by LHCII immobilized within porous graphite electrode (GE) might depend on endogenous PQ molecules, non-specifically bound to complexes^12^. In this report, we verify the operation of a graphite electrode-based biohybrid system, in which we use LHCII_3_ as the photoactive layer and PPBQ as the MET reagent. The choice of synthetic quinone has relied on the properties of these compounds in quenching Chl fluorescence and electron transport capabilities in biophotovoltaic systems. Our results show that the combination of these properties enables the design of an efficient photocurrent generating system. Furthermore, our study shows that LHCII freely migrates into the interior of the porous GE, as well as that the PPBQ redox reaction proceeds more efficiently in the presence of immobilized LHCII_3_. We have based our conclusions on measurements of Chl fluorescence quenching, PPBQ light reduction, voltammetry, and electrochemical impedance spectroscopy as well as analysis by fluorescence microscopy in situ. Finally, our work highlights the ability to use LHCII in connection with quinone derivatives in a biophotovoltaic device based on the carbon electrode.

## Results and discussion

**Figure 1:**
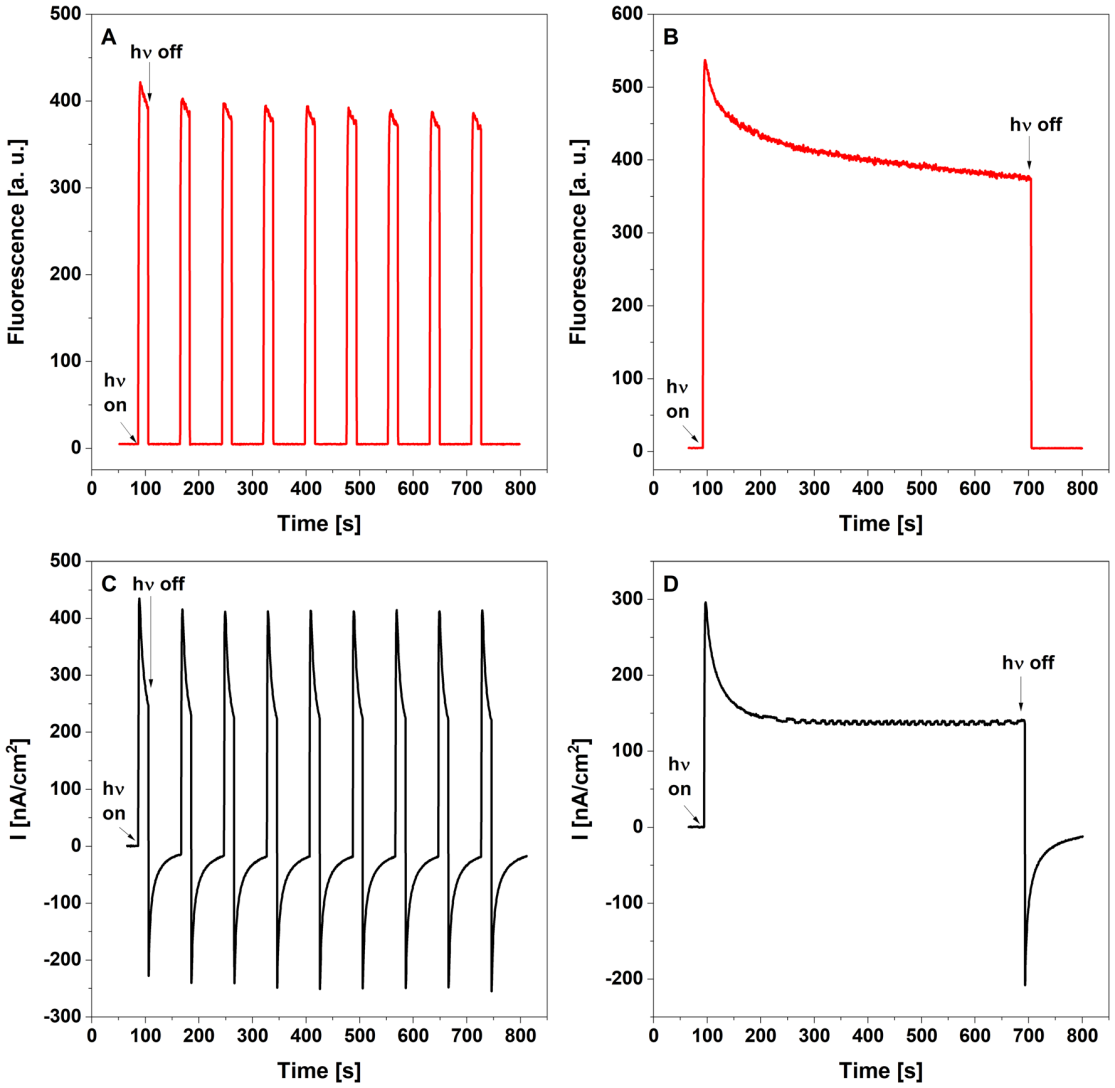
Simultaneous measurements of fluorescence emission and photocurrent generation in GE-LHCII_3_-PPBQ system. Measurements were carried out under dark/light cycles (**A**, **C**) as well as continuous illumination (**B**, **D**). The fluorescence traces were presented as red lines (**A**, **B**) whereas the respective photocurrent response as black lines (**C**, **D**). The sample was excited with the actinic blue light intensity of 4900 µE. The arrows show the points of switching on and off the actinic light. The presented data are representative of at least 10 separate experiments.

Photocurrent generation and Chl fluorescence from GE-LHCII_3_ in the presence of 0.1 mM PPBQ were detected simultaneously under the 20 s pulse (Fig. 1A) and continuous illumination (Fig. 1B). The illumination of this GE-LHCII_3_-PPBQ system with the blue light of 4900 μE followed the 60 s dark period induced a rapid, in the 4 s range, increase of fluorescence emission at 680 nm (Fig. 1A,B). At the same time, in the 2 s range, the anodic photocurrent was growing to maximum levels in both experimental conditions (Fig. 1C,D). Although both processes underwent limited quenching (Fig. S2), the maxima of anodic photocurrent were invariable under the nine dark/light cycles and, except for the 60 s declining period, under continuous illumination (Fig. 1D). This quenching phenomena is probably related to transient processes in the diffusion layer at the GE surface^30^.The Chl fluorescence, but not the photocurrent, completely declined when the light was turned off. Beginning from the dark period, the opposite cathodic current was noted (Fig. 1C,D), which in the minimum point abruptly changed the direction towards the anodic one and hyperbolically increased up to the initial value (Fig. 1C,D). Although photocurrent was generated in the GE-LHCII system without exogenous redox mediators^12^, the addition of the PPBQ to the reaction buffer caused a 20-fold increase in photocurrent intensity (Fig. S3A), whereas the release of PPBQ from the reaction vessel decreased this reaction (Fig. S3B).

**Figure 2:**
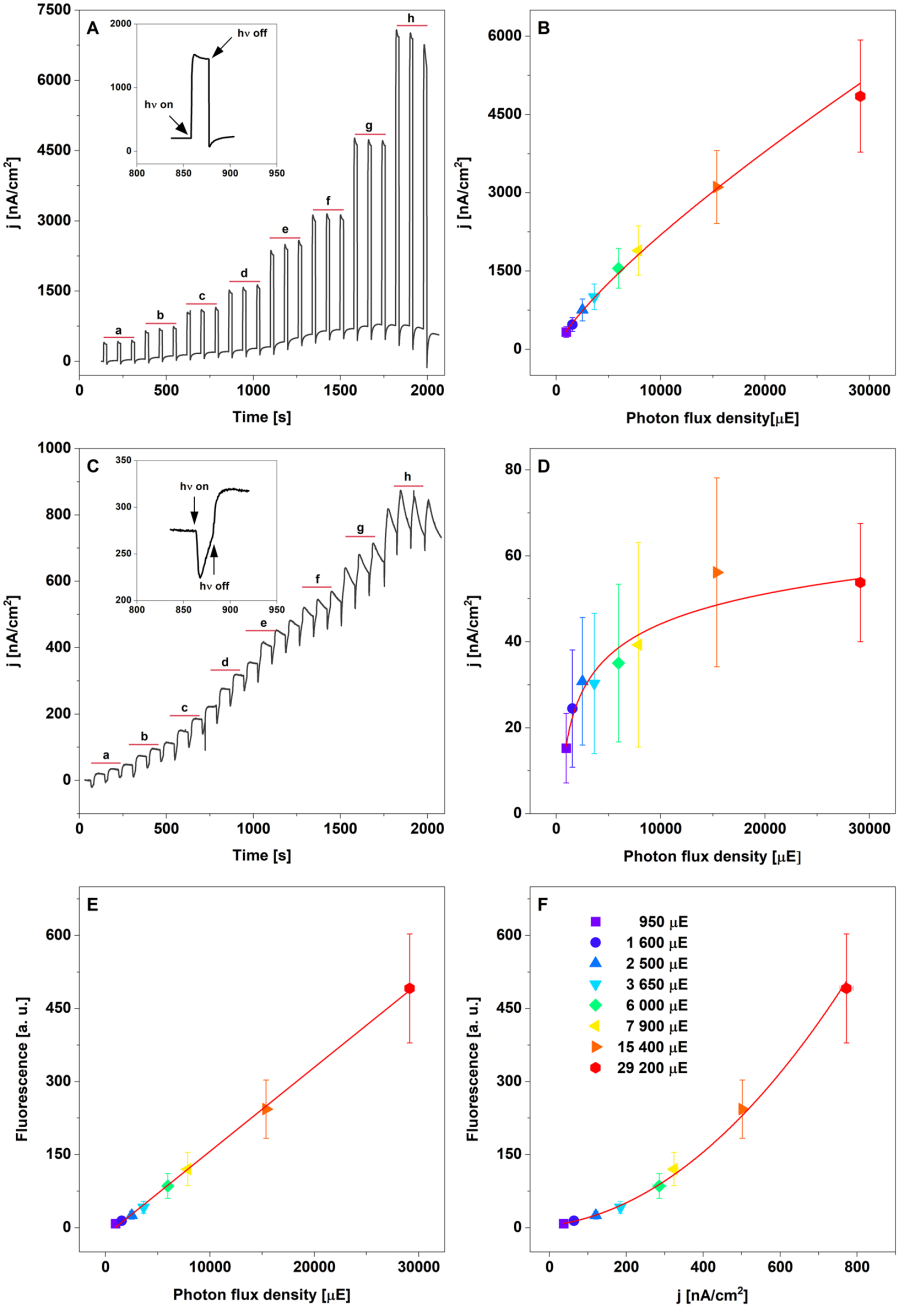
Response of GE-LHCII_3_-PPBQ and GE-PPBQ systems to a gradual increase of light intensity. Panels **A** and **C** show the changes of current under the dark/light cycle, where the insets (**A**, **C**) show the turned points for these cycles (20 s light/60 s dark). Panels **B** and **D** present the relation between anodic and cathodic current density and photon flux density (PFD) for GE-LHCII_3_-PPBQ and GE-PPBQ, respectively. In panels **E** and **F** the relationship of fluorescence and current density, respectively versus PFD for GE-LHCII_3_-PPBQ were calculated. The actinic white-light was used for induction of anodic (**A**, **B**) and cathodic (**C**, **D**) photocurrents whereas for the simultaneous induction of Chl fluorescence and anodic photocurrent the actinic blue-light was used (**E**, **F**). The letters (a–h) in panels **A** and **B** indicate the actinic light intensity from 950 (a) to 29 200 µE (h), in the order presented in panel **F**. The non-linear fitting curves were calculated for average data (solid red lines). The data are mean values ± SD for 5 independent experiments.

We have investigated the generation of current in the function of light intensity for two experimental conditions, (i) the system contained LHCII_3_ and PPBQ (GE-LHCII_3_-PPBQ) and (ii) solely for redox mediator (GE-PPBQ) (Fig. 2). The illumination of GE-LHCII_3_-PPBQ with gradually increasing photon flux density (PFD) of actinic white light caused the near-linear increase of the anodic photocurrent up to about 4.5 μA/cm^2^ (Fig. 2A,B). Arising simultaneously dark current revealed a relatively low amplitude and was dependent on light intensity (Fig. 2A). The illumination of GE-PPBQ with the gradually increasing intensity of white light caused a rapid generation of the cathodic current, which revealed the hyperbolic relation to light intensity (Fig. 2C,D). However, the light-dependent flow of current in the GE-PPBQ system was more complicated. The cathodic current after 4–6 s rapidly changed the direction towards the anodic one (Fig. 2C). The successive dark/light cycle caused an increase of the overall electrochemical potential of the GE-PPBQ system, what can suggest a gradual shift in the ratio of various radical forms of PPBQ, with different redox properties, as described for other quinone molecules^31^. In the GE-LHCII_3_-PPBQ system, the course of Chl fluorescence was linear in relation to PFD (Fig. 2E) and exponential concerning photocurrent generation (Fig. 2F), indicating that at higher PFD relatively more absorbed energy is dissipated than converted to the photocurrent.

**Figure 3:**
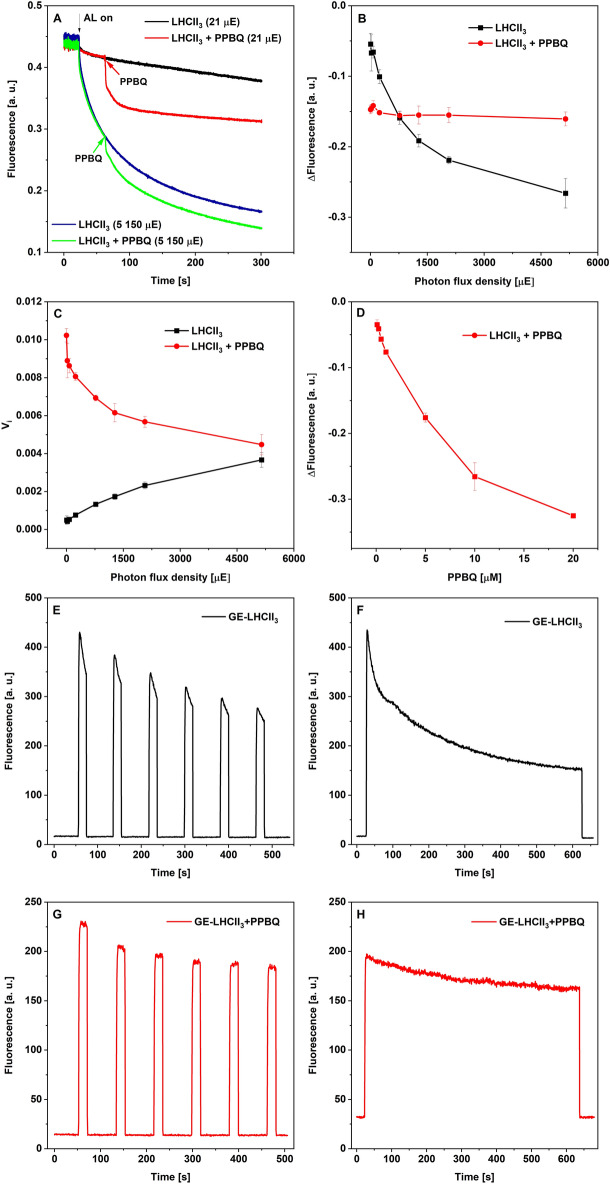
Chl *a* fluorescence quenching induced by light and PPBQ measured in LHCII_3_ solution with means of PAM fluorimeter (**A**–**D**) and in GE-LHCII_3_ system with the use of special holder (Schematic diagram S1) (**E**–**H**). Panel **A** shows representative Chl *a* fluorescence quenching traces measured for LHCII_3_ solution (50 µg/ml). The arrows show the points when the actinic light (AL, black) or PPBQ (20 µM) were applied, respectively. Panel **B** shows the overall decrease of Chl fluorescence in relation to PFD in the absence (black) or presence (red) of 20 µM PPBQ. The values were calculated as a difference between fluorescence in the initial point of reaction (light switch-on for LHCII_3_ alone or addition of PPBQ for LHCII_3_ + PPBQ) and fluorescence level after 300 s of illumination. Panel **C** shows the initial velocity of quenching (V_i_) calculated for the same data as presented on panel **B**. The V_i_ values were calculated from the initial rectilinear part of the fluorescence curve. Panel **D** presents the fluorescence change recorded at different PPBQ concentrations. The quenching traces were induced by actinic light with the same intensity (21 µE) and PPBQ was present in solution from the beginning of the reaction. The values were calculated as a difference between the initial and final point of the fluorescence curve. The data presented in panels **B**–**D** are mean values ± SD for 3 independent experiments. Panels **E**–**H** show fluorescence emission from GE-LHCII_3_ in the absence and presence of 0.1 mM PPBQ under dark/light cycle (**E**, **G**) and continuous illumination (**F**, **H**). Fluorescence emission at 680 nm was measured under applying OCP and actinic blue light with the intensity of 4900 µE and is representative of 3 separate experiments.

Analyses of light-dependent Chl fluorescence quenching in the presence of PPBQ were performed under two experimental conditions, using the LHCII_3_ solution (Fig. 3A–D) and the GE-LHCII_3_ system (Fig. 3E–H). The overall Chl *a* fluorescence of LHCII_3_ in the absence of PPBQ decreased proportionally to PFD (Fig. 3B), and the initial velocity of quenching (V_i_) increased with rising light intensity (Fig. 3C). Otherwise, the overall decline of fluorescence in the presence of 20 µM PPBQ was independent of light intensity (Fig. 3B). However, the initial quenching velocity in the presence of PPBQ (V_i+PPBQ_) decreased proportionally to PFD and revealed the opposite direction to V_i_ (Fig. 3C). It should be noted that the sum of V_i_ and V_i+PPBQ_ for successive points of light intensities was practically invariable taking roughly 0.0084 fluorescence unit s^–1^, suggesting that the total velocity of fluorescence quenching, induced by light and PPBQ, is impassable at a particular concentration of LHCII_3_. The quenching of fluorescence measured at stable illumination (21 µE) and increasing concentration of PPBQ reached saturation at its concentration slightly above 20 µM (Fig. 3D). These data imply that the PPBQ is an efficient excitation quencher which impacts proportionally to the number of accessible quenching sites in LHCII_3_, what is in agreement with previous observations^22^.

The decline of fluorescence of GE-LHCII_3_ under continuous illumination was similar to the quenching detected for LHCII_3_ solution at the same PFD (Fig. 3F), whereas the fluorescence quenching observed for dark/light cycles was about one-half smaller, probably due to partial reversal of light-related quenching effect after each period of darkness (Fig. 3E)^32^. The addition of 0.1 mM PPBQ to the GE-LHCII_3_ system decreased the initial fluorescence levels by about one-half, but the subsequent illumination deepens the quenching effect only by about 20% (Fig. 3G,H). These indicate that the total fluorescence quenching of GE-LHCII_3_ is dependent on the combined effect of PPBQ concentration, light intensity, and accessible quenching sites in LHCII_3_. Furthermore, a comparison of fluorescence quenching of LHCII_3_ solution (Fig. 3B) and GE-LHCII_3_ (Fig. 3H) indicates that the concentration of 0.1 mM PPBQ applied for the generation of photocurrent (Fig. 1,2) was used above the saturation.

The PPBQ-dependent fluorescence quenching of LHCII_3_, as well as participation in energy transfer from LHCII_3_ toward GE, require close contact between PPBQ and LHCII_3_. Because this interaction could influence the electrochemical properties of the biohybrid system, we performed series of voltammetric analyses. The differential pulse voltammetry (DPV) analyzes showed that the oxidation and reduction peaks for PPBQ were significantly far away from peaks for LHCII_3_ (around + 0.500 V and − 1.200 V) (Fig. S4).

**Figure 4:**
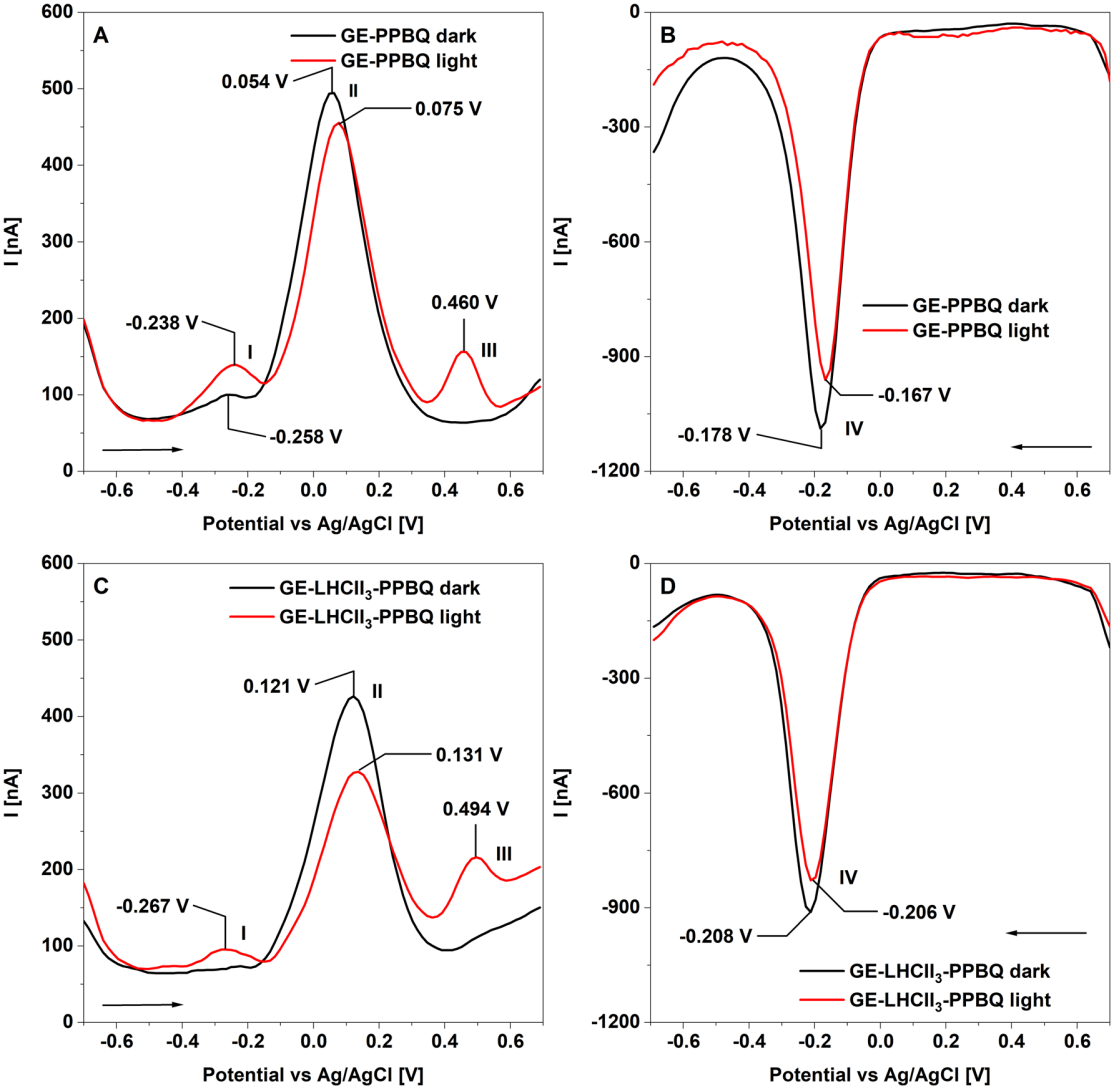
Voltammetric characterization of the interaction between LHCII_3_ and PPBQ. Differential pulse voltammograms (DPV) of GE-PPBQ (**A**, **B**) and GE-LHCII_3_-PPBQ (**C**, **D**) for oxidation (**A**, **C**) and reduction (**B**, **D**) processes were recorded under dark (black lines) and light (red lines) conditions. The measurements were performed in special holders and samples were illuminated with actinic white light with the intensity of 4900 µE. PPBQ was used at 0.1 mM concentration. The arrows indicate the direction of the potential change. Curves were smoothed using a polynomial fixed order available through NOVA software. The presented voltammograms are representative of at least 3 separate experiments.

The DPV analyzes of PPBQ in a narrower potential range under dark conditions revealed a small anodic peak I at around − 0.260 V and a higher peak II at roughly + 0.055 V (Fig. 4A). The sample illumination caused the appearance of an additional peak III around + 0.460 V (Fig. 4A). The statistical analyses (Table 1) showed that the potential of peak I is not dependent on light conditions, whereas the potential of peak II significantly shifted towards higher values under illumination (+ 0.074 V). Otherwise, the cathodic peak IV (− 0.180 V) shifted to more positive values (− 0.160 V) (Fig. 4B, Table 1). Similar data were obtained using cyclic voltammetry (CV) (Fig. S5B) and square wave voltammetry (SWV) (Fig. S6A,B, Table S1). The absorption spectra of the illuminated PPBQ solution also showed the formation of new reduced forms of this compound (Fig. S5A).

**Table 1:** Average potentials of peaks at the voltammograms registered for oxidation and reduction processes of GE-PPBQ and LHCII_3_-GE-PPBQ under dark and light conditions.

Peak number	Differential pulse voltammetry (DPV)			
	I (V)	II (V)	III (V)	IV (V)
*Experimental systems*				
GE-PPBQ dark	− 0.260 ± 0.019	0.055 ± 0,002^a,c^	–	− 0.180 ± 0.003^a,c^
GE-LHCII_3_-PPBQ dark	–	0.114 ± 0.020^a^	–	− 0.210 ± 0.013^a^
GE-PPBQ light	− 0.239 ± 0.002	0.074 ± 0.010^b,c^	0.462 ± 0.004^b^	− 0.160 ± 0.009^b,c^
GE-LHCII_3_-PPBQ light	− 0.251 ± 0.019	0.134 ± 0.011^b^	0.484 ± 0.013^b^	− 0.202 ± 0.013^b^

The average values ± SD were calculated from 4 to 7 independent experiments. Values marked with letters indicate a significant difference (*p* < 0.05) between GE-PPBQ and GE-LHCII_3_-PPBQ measured under dark (^a^) or light (^b^) conditions as well as between dark and light measurements for GE-PPBQ (^c^).

The DPV scans of GE-LHCII_3_-PPBQ under dark conditions revealed the dominated anodic peak II at roughly + 0.120 V and cathodic peak IV (− 0.210 V) (Fig. 4C,D, Table 1). The light conditions induced the appearance of the additional anodic peaks I and III (− 0.270 and + 0.490 V) on the DPV scans, whereas the reduction peak IV was like that detected under the dark conditions (Fig. 4C,D, Table 1). The main voltammetric differences between GE-PPBQ and GE-LHCII_3_-PPBQ ones were presented in DPV as well as in SWV scans as a shift of the maximum of the peak II by about 0.060 V toward more positive potential (Figs. 4. Fig. S6, Table 1). Significant potential shifts were also shown for anodic peak III and cathodic peak IV, toward the more positive or more negative values (Table 1), respectively. These changes were observed both for dark and light conditions.

The data presented above proved that the specific interaction between PPBQ and LHCII_3_ is necessary to initiate the anodic current flow (Figs. 1, 2, 3, 4). Simultaneously, we found that in the GE-PPBQ system the illumination induced a cathodic photocurrent, which changed to the anodic in a few seconds (Fig. 2C). Unexpectedly, this phenomenon is observed under a dark gap during photocurrent generation in the GE-LHCII_3_-PPBQ system (Fig. 1C). Analyzes of absorbance spectra during photochemical reactions revealed that light-induced reduction of PPBQ occurred in the buffer phase independently on photocurrent generation (Fig. S7) and that the alternating photocurrent flow is likely related to the rate of diffusion of reduced forms of PPBQ into GE (Fig. S7I). Quinones undergo redox processes including reactions of single electron transfer, proton-coupled electron transfer and hydride transfer. These lead to the formation of a number of molecular forms of quinones, as well as radical intermediate compounds. Quinone photoactivation changes their redox potentials, improves their ability to accept hydrogen, and increases their reactivity^31,33^**.** Thus, observed light-induced shift of the PPBQ redox potentials (Figs. 4, S6, Table 1) indicate the formation of PPBQ forms with higher reactivity, and only these forms take up or give up electrons to the GE (Figs. 2C, S7I).

The quenching property of PPBQ was confirmed in our study both in solution and in GE-LHCII_3_-PPBQ system (Fig. 3). The quencher can interact with Chl molecule through a dynamic or static mechanism. The first one occurs through collisions with excited Chl molecules, whereas in the second one the quencher forms a complex with a Chl in the ground state^22^. Our voltammetric analysis of GE-LHCII_3_-PPBQ system revealed the significant shift of redox potentials regarding to GE-PPBQ both under dark and light conditions (Figs. 4, S6, Table 1), which can indicate the formation of complexes between PPBQ and Chl in ground as well as in excited states. This assumption can be substantiated by the previously observed ground-state interactions between chlorophyll analogue molecule, zinc chlorin Zn*Ce6* and benzoquinone, where Zn ion bound in the *Ce6* moiety was required for complex formation^34^. Furthermore, the quenching effects of quinones on excited states of Zn*Ce6* were analyzed in solution as well as with use of peptide maquette, where peptide scaffold ensures stabilization of the pigment and therefore higher quenching efficiency^34–36^. The proposed quenching scheme assumes that formation of the coupled radical pair of the pigment cation and quinone anion (P^·+^Q^·−^) by charge-transfer reaction leads to quenching of the excited pigment P*. In the next step, the radical pair can be separated into pigment radical and quinone radical (P^·+^ + Q^·−^), what makes possible further electron transfer reactions to other molecules by charge separation reactions^34^.

Questions can be raised whether a similar phenomenon could occur in our experimental GE-LHCII_3_-PPBQ system, namely (i) formation of P^·+^Q^·−^ radical pairs and (ii) the further electron transfer by the charge separation mechanism leading to photocurrent generation. (Figs. 1, 2). The Chls and carotenoids in LHCII are precisely oriented to maximize light-harvesting efficiency and transfer energies to a Chl *a* in terminal emitter domain of Chls 610/611/612 or to Chl *a* 604, the alternative energy sink^23,24^. Considering that these domains might be sites of the efficient energy transfer to quinones, the theoretical possibility of specific binding of PPBQ to Lhcb1 protein was checked. Our computer models obtained with ROSIE software indicated the plausible binding of PPBQ with Lhcb1 protein in the domains corresponding to the sites of binding of these terminal Chls (Fig. S8C,D), which makes the formation of a Chl-PPBQ complex probable.

The currently proposed model for dissipation of excited state in isolated LHCII assumes that this process is located in domain of Chls 610/611/612 along with its associated conjugated amino acids (aa) residues. This model predicts the formation of Chl–Chl charge-transfer states as quenching intermediates and sequence of proton-coupled electron transfer steps involving associated aa^37,38^. Our computer model (Fig. S8C) indicates that PPBQ can be bound by 178–183 aa residues, close to this quenching domain. Considering the efficient quenching properties of PPBQ (Fig. 3), voltammetric data (Fig. 4) and structural model, it is very likely that redox complexes arise in the terminal emitter domain of LHCII. Since PPBQ can bind to aa residues involved in quenching process^37^, it is possible that these aa residues involved in the formation of redox-active complexes. The potential of the exciting quinones undergoes changes due to the ground state, what causes easier reduction or oxidation those molecules^18,33^. Thus, in the case of PPBQ, the light-induced quinone forms (Fig. 4, Table S1) might be active components of the quenching state, created directly or indirectly with excited Chl.

The PQ are specifically bound to domains inside the PSII protein scaffold and the polarity of aa residues surrounding bounded mediator supports the redox properties of quinones and favors further electron transfer^16^. In the case of PPBQ and LHCII_3_ interactions, our computer model shows that PPBQ is bound more superficially to the protein and exposed to aqueous environment (Fig. S8). In aqueous solution, the equilibrium between Q^·−^ and conjugated semiquinone radical HQ^·^ is established, which may prevent the feedback reaction^34^. Thus, it is possible that the PPBQ radical, formed as a result of Chl quenching (Fig. 4), interacts with the water molecules and undergoes protonation, which may favor charge separation in LHCII_3_-PPBQ system. At the PSII core complex, the electrons from the reaction center are transferred to PQ bounding in Q_B_ site, where the stable semiquinone radical ${\mathrm{Q}}_{\mathrm{B}}^{\bullet -}$ is formed, which undergoes protonation to Q_B_H_2_. The diffusion of Q_B_H_2_ from the Q_B_ site is thermodynamically favorable, providing efficient exchange of the PQ/PQH_2_ pool^39^. It can be assumed that the protonated quinone could diffuse toward the GE in the system of LHCII-PPBQ, starting the current flow (Figs. 1, 2).

**Figure 5:**
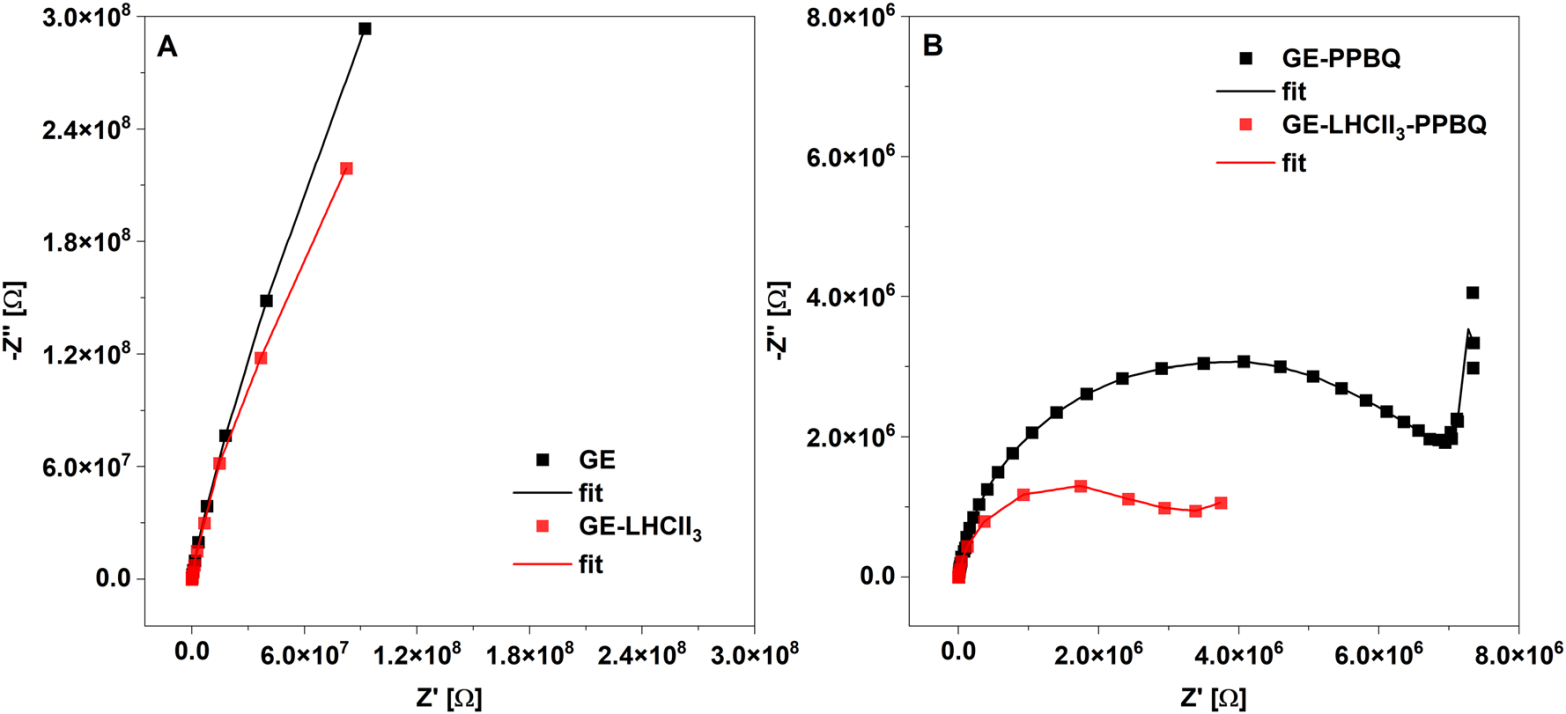
Electrochemical impedance spectroscopy (EIS) spectra recorded for each modification step of the graphite electrode. Nyquist plots for: unmodified GE electrode in HEPES buffer (black squares) and GE in the presence of 0.1 mM PPBQ (GE-PPBQ)(red squares) (**A**), GE modified by 1.5 µl suspension of LHCII_3_ in glutaraldehyde matrix (0.2 µg Chl/µl) (LHCII_3_-GE) (black squares) and final biohybrid system GE-LHCII_3_-PPBQ (red squares) (**B**). The EIS spectra were recorded under dark conditions at OCP. The plots are representative of 3 separate experiments.

However, we found the formation of Chl-PPBQ redox complexes in the ground as well as in the excited state (Fig. 4, Table 1), but the duration time of these complexes was not determined. Thus, it cannot be excluded that these complexes are stable and that there is no diffusion of a protonated form of PPBQ. Simultaneously, the light induced formation of excited/reduced forms of PPBQ in buffer phase (Figs. 4, 5) and LHCII-independent current flow occurs (Figs. 2, 5). Besides that, the short-lived cathodic dark-current is probably related to the establishment of equilibrium between the different redox forms of PPBQ (Figs. 1, S7). This suggests that PPBQ reveals dual action, while some molecules bind to the LHCII_3_, and form complexes involved in quenching reactions, and others transfer the charge and enable photocurrent to appear. Since the value of the photocurrent generated by the GE-PPBQ system is ten times smaller than that by the GE-LHCII_3_-PPBQ (Fig. 2), there must be a coupling point between the bound and free PPBQ that allows efficient transfer of the light energy harvested by LHCII_3_. However, the interaction of Chl-PPBQ complexes with the electrode by the DET mechanism cannot be dismissed either. The exact solution to these issues will be possible after applying time-resolved measurement methods enabling to investigate the excited states of Chl and quinones^18,34,37^. Regardless of the need for a detailed explanation of the system's operation, our GE-LHCII_3_-PPBQ system efficiently generates a photocurrent proportional to the light intensity up to the value of about 5 μA/cm^2^ (Figs. 1, 2). The internal quantum efficiency (IQE) calculated for blue light as was described in^40^ and estimated to 0.66% indicates an efficient charge separation, also compared to other biohybrid systems^40,41^.

**Figure 6:**
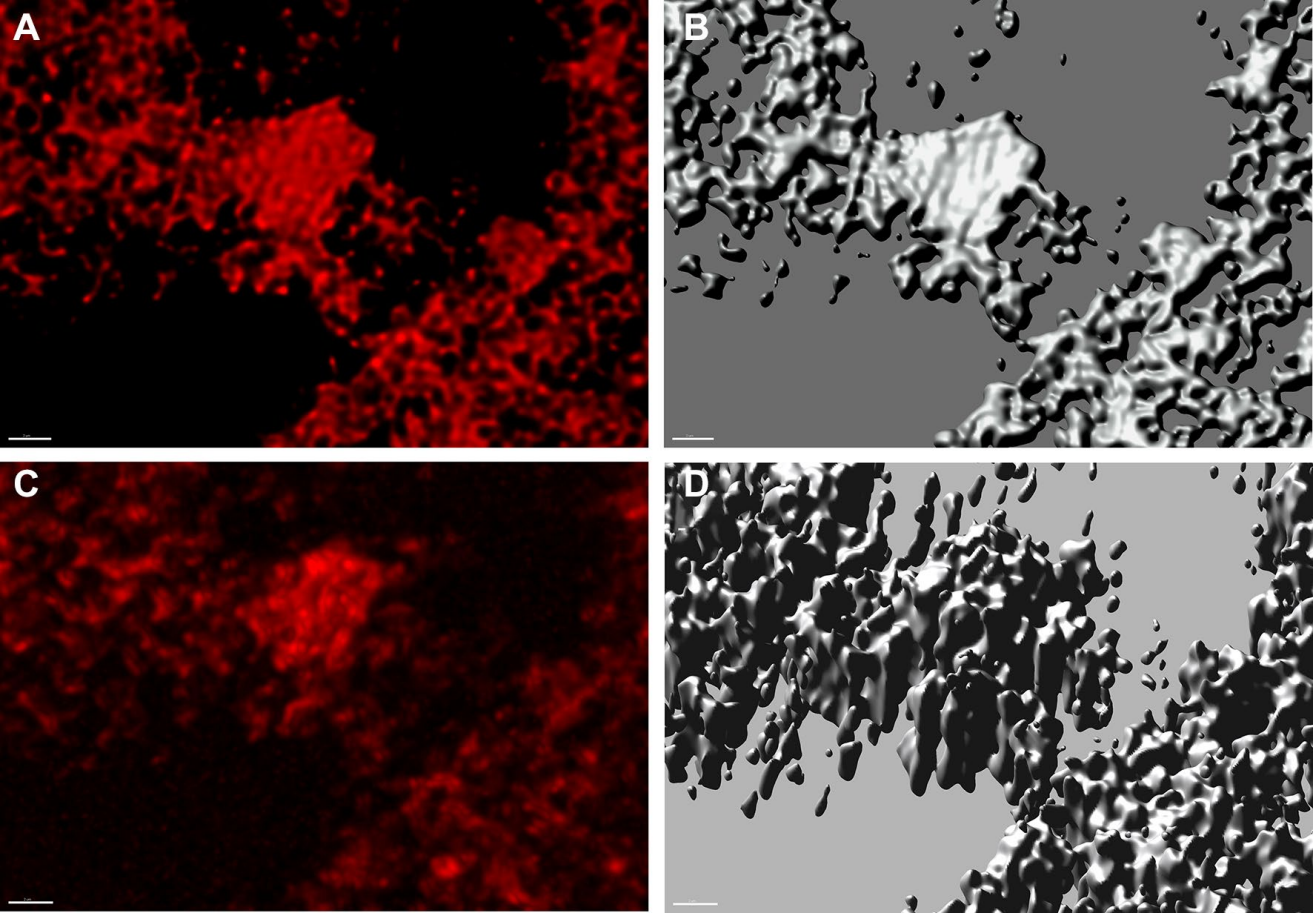
Confocal laser scanning microscopy (CLSM) imaging of Chl fluorescence of LHCII_3_ from the same area of GE-LHCII_3_-PPBQ before (**A**, **B**) and after 20 min of OCP treatment (**C**, **D**). The images were registered with the use of a special holder (Schematic diagram S2) for CLSM. For better visualization of the 3D distribution of LHCII_3_ inside GE the area incompletely covered by complexes was selected. The left panel (**A**, **C**)—top view of the 3D distribution of red Chl fluorescence, the right panel (**B**, **D**)—the computer-generated 3D reconstruction of the fluorescence surface presented at a 10° angle.

Regardless of charge separation efficiency, the effectiveness of the biohybrid system is attributed to active electrode surface area, which affects both the DET and MET response^7–10^. Therefore, we checked the electrochemical processes occurring in the vicinity of the GE by Electrochemical Impedance Spectroscopy (EIS). At the applied potential, no redox processes were observed on the empty GE immersed in the buffer and on the GE covered by LHCII_3_ in glutaraldehyde matrix (GE-LHCII_3_), as presented by no semicircle part in the Nyquist plots and no peak in the Bode phase plots (Figs. 5A, S9C). The relatively low double-layer charging (Table 2) can prove the LHCII_3_ migration into pores of the graphite electrode and its electrostatic stabilization. In the presence of PPBQ the semicircles at the Nyquist plots (Fig. 5B) and peaks at Bode phase plots (Fig. S9B,D) appeared. The values of double-layer charging increased more than 10 times for GE-PPBQ and more than 100 times for GE-LHCII_3_-PPBQ comparing to systems without PPBQ (Table 2). Furthermore, a lower charge transfer resistance was observed in the GE-LHCII_3_-PPBQ than in the GE-PPBQ (Fig. 5B vs. A and Table 2). This means that in the presence of LHCII_3_, PPBQ underwent a redox reaction more easily, so in GE-LHCII_3_-PPBQ the electron transfer was more efficient, and the conductivity improved. Furthermore, the linear part of the Nyquist plot for GE-LHCII_3_-PPBQ indicated that in the presence of LHCII_3_ increased diffusion of PPBQ between the electrical double layer and the buffer phase (Fig. 6B) occurred.

**Table 2:** Parameters obtained by fitting EIS spectra to an electrochemical model.

Experimental systems	Charge transfer resistance (R_ct_) [kΩ]	Double layer charging (C_dl_) [nMho*s^N^]
GE	28.57	21.70
GE-PPBQ	6.87	323.56
GE-LHCII_3_	65.27	4.03
GE-LHCII_3_-PPBQ	2.29	416.90

Independently on electrochemical methods, the properties of the electrode surface were directly investigated by confocal laser scanning microscopy (CLSM) and scanning electron microscopy (SEM). Using the CLSM and a special holder (Scheme S2), we were able to obtain successive optical cross sections of GE-LHCII_3_-PPBQ and determine changes in the three-dimensional (3D) position of the LHCII_3_ inside GE during in situ experiments. LHCII_3_ deposited on GE surface before electrochemical treatment was shown as flat, partially undulated layers with 3–6 μm thickness (Fig. 6A,B), suggesting that the LHCII surface covering the GE was composed of many layers, where neighboring LHCII_3_ are located close to each other. Imaging of Chl fluorescence from the same area after 20 min of OCP treatment under the dark conditions revealed that the density of fluorescence in the single optical layer decreased, and fluorescence was recorded in a larger number of layers localized deeper in the GE structure (Fig. 6C). The 3D models indicated that the changes in fluorescence localization occurred in both vertical and horizontal directions (Fig. 6B versus D).

We are no longer dealing with the flat multilayer surface, but with the irregular 3D structure with 16–22 μm thickness (Fig. 6D). The total surface area (A, μm^2^) and volume (V, μm^3^) of 3D models were roughly 5 and 9 times higher, respectively, after OCP treatment and the A/V ratio decreased from 3.6 to 2.0, which is typical for the increasing volume of ellipsoid-like structures. Observed by CLSM in situ migration of LHCII_3_ agreed with SEM images, where the disappearance of the surface layer of LHCII_3_ after OCP treatment was observed (Fig. S10C). The fluorescence spectra recorded in different depths of GE-LHCII_3_-PPBQ revealed the same shape with a maximum of around 684 nm (Fig. S11), similarly to room temperature spectra of isolated LHCII^42^. The signal of free Chl at 665 nm was not noted^12^, that confirms retaining of LHCII_3_ in its native structure. These observations point to quite unlimited migration of LHCII_3_ into GE with concomitant decreasing of surface-density of complexes.

The LHCII was so far applied in the construction of DSSC as a photosensitizing layer on TiO_2_/FTO surface with the use of ${\mathrm{I}}_{3}^{-}$/3 ${\mathrm{I}}^{-}$ as a MET reagent. The operation of such systems assumes that the excited state of Chl introduces electrons into the conduction band of TiO_2_, while the operation of the ${\mathrm{I}}_{3}^{-}$/3 ${\mathrm{I}}^{-}$ cycle fills the electron gap created by charge separation in the Chl molecule. These systems have revealed high stability and long operation times. However, the charge regeneration occurs with low efficiency and the mechanism of this process has been not explained yet^26–28^. Our biohybrid system operates by a different mechanism based on the effect of quenching of excited states of Chl combined with the MET mechanism, both of which depend on the presence of exogenous quinone.

The choice of graphite as a solid phase was dictated not only by its electrochemical properties and porous structure but also by the prospects associated with the development of carbon nanomaterials. Diffusion of LHCII_3_ into porous GE leads to a reduction in the density of the complexes (Fig. 6) and decreases the probability of excitation quenching by complex aggregation^22^. The relatively low charge transfer resistance for GE-LHCII_3_-PPBQ suggests that LHCII_3_ did not form tight multilayers at the electrode surface (Table 2). The redox reactions involving PPBQ as well as diffusion of PPBQ increased in the presence of LHCII_3_ (Fig. 5, Table 2), which would support the assumption of a PPBQ pool linking quenching reactions with electron transfer toward GE. Both microscopic and EIS analyses suggest bounding of LHCII_3_ to exposed surfaces of the graphite scraps, which leads to the formation of a large 3D active surface area.

## Conclusions

The combination of three elements (i) the trimeric (LHCII_3_) antenna complexes, (ii) the porous graphite electrode (GE) and (iii) the application of PPBQ, the quinone derivative, which acts as a quencher of excited Chl in LHCII_3_ and as an electrochemical mediator, allows the construction of a new type of biohybrid photoactive system (GE-LHCII_3_-PPBQ). This system has generated current proportional to a wide range of light intensity and has shown an internal quantum efficiency of at least 0.66%. The three-dimensional arrangement of the graphite scraps in GE ensured that LHCII_3_ was bound throughout the electrode volume and stabilized in its native form on graphite surface probably in low-density multilayers. The primary excitation quenching reaction by PPBQ is associated with the formation of Chl-PPBQ redox complexes, both in the ground and excited states. The inference is complicated by the fact that PPBQ is a photoactive chromophore independently generating photocurrent. Thus, it can be assumed that coupling of LHCII_3_ and PPBQ photocycles causes a rise of photocurrent generation in GE-LHCII_3_-PPBQ system. Our research indicates the possibility of using antenna complexes in connection with quinone derivatives and carbon electrode in construction of useful biophotovoltaic device. Further research on the use of quinone derivatives with higher affinity to LHCII and carbon materials enabling enlargement of the active surface of the electrode should lead to increased performance of this biohybrid system.

## Methods

### Isolation of LHCII complexes

LHCII was isolated from spinach leaves purchased at the local market according to the protocol described previously^43^. The isolated LHCII complexes were suspended in 20 mM Tricine buffer (pH 7.8) and stored at – 80 °C until use. The purity of the preparation has been tested using SDS-PAGE electrophoresis and immunodetection. The analyzes showed the presence of Lhcb1, Lhcb2, and Lhcb3 polypeptides and the lack of the D1 and D2 of PSII core subunits (Fig. S1), which indicates the high purity of the LHCII samples used according to our previous results ^12^.

### Screen-printed graphite electrode (GE) and pretreatment procedure

All electrochemical experiments were carried out using a disposable planar screen-printed three-electrode system composed of a round graphite working electrode (geometric area of 0.07 cm^2^), an Ag/AgCl pseudo-reference electrode (Ag/AgCl RE), and a graphite counter electrode. The properties of this graphite electrode (GE), as well as the procedure of cleaning and activation of the carbon surface, were described in details previously^12,44^. The potential of a pseudo-reference electrode in HEPES buffer (20 mM HEPES, 15 mM NaCl, 4 mM MgCl_2_, pH 7.5) was controlled against the saturated calomel electrode (SCE) and was equal to 0.080 ± 0.005 V. This gives a potential equal to 0.324 ± 0.005 V against a normal hydrogen electrode (NHE). All potentials reported in this study are given in comparison to the Ag/AgCl RE.

### LHCII trimers (LHCII_3_) and GE-LHCII_3_ preparation

A trimeric form of LHCII was formed by the addition of n-Dodecyl-β-D-maltoside (DDM) to freshly defrosted LHCII, at a final concentration of 0.025% (w/v). The trimeric state was confirmed by analyses of Chl fluorescence spectra at 77 K (Fig. S1) showing the typical fluorescence emission bands^45^. The LHCII_3_ was mixed with glutaraldehyde to the final concentration of 0.2 µg Chl/µl and 0.75% (w/v) glutaraldehyde and 1.5 µl of the final suspension was immediately deposited on the graphite surface of the working electrode (GE) and followed dried at room temperature for 15 min to form a layer of LHCII_3_-glutaraldehyde. Then, after immersion in HEPES buffer and determination of the open circuit potential (OCP), the electrode was exposed to this potential for 1 min, what finally resulted in forming GE-LHCII_3_ biohybrid system. The LHCII_3_ preparation procedures were performed under diffuse green light whereas OCP values were determined in the dark.

### The electrochemical and fluorescence measuring system

A holder, designed especially for our experiments, enables placement of a glass cuvette within GE in a closed box with three sockets connected by the optical fibers with the actinic light source and spectrofluorimeter (Schematic diagram S1). The GE was connected with AUTOLAB PGSTAT 128 electrochemical analysis system equipped with NOVA 2.1 software package (EcoChemie, The Netherlands). The measuring part of GE was immersed in HEPES buffer saturated with argon. Actinic light was achieved with the Schott KL 2500 LCD light source with an intensity from 950 to 29 200 µE. The photosynthetic photon flux density (given as µmol photons s^–1^ m^–2^; µE) was measured using a Universal Light Meter (ULM-500, Heinz Walz GmbH, Effeltrich, Germany).

For simultaneous detection of Chl fluorescence and photocurrent response, the fluorescence of the GE-LHCII_3_ in the presence of PPBQ was measured with the Shimadzu RF-5301PC spectrofluorimeter with 5 nm spectral resolution for excitation at 470 nm and emission at 680 nm. The actinic blue light (377–504 nm) was used, and the emission was recorded through LP600 filter to remove the influence of actinic light. The actinic white light (280–800 nm) was applied to induce the photocurrent itself, due to overlapping the red regions of spectra of actinic light and Chl *a* fluorescence emission.

The OCP established to roughly –0.150 V was applied in all experiments. Cyclic voltammetry (CV) was performed in potentiostatic mode, and the CV curves were recorded at the scan rate of 0.1 V s^–1^. Differential pulse voltammetry (DPV) and square-wave voltammetry (SWV) were performed under illumination and under dark conditions within the scan range from 0.3 to – 1.5 V and from – 0.7 to 1.0 V (versus Ag/AgCl) to monitor reduction and oxidation processes, respectively. Electrochemical impedance spectroscopy (EIS) was performed at a frequency range from 10^5^ to 10^–3^ Hz to measure the internal resistance. Nyquist graphs were plotted using NOVA software. EIS measurements were conducted in the darkness in a homemade Faraday cage.

### Chl *a* fluorescence and spectroscopy measurements

Chlorophyll *a* fluorescence quenching was measured in a solution of LHCII_3_ in the presence or absence of PPBQ by the Pulse-Amplitude-Modulation approach using the Dual-PAM 100 (Heinz Walz GmbH, Effeltrich, Germany). The excited modulated blue light (460 nm) with intensity below 2 μE and detection above 700 nm (long-pass RG9 filter) was applied to establish initial measuring conditions. Fluorescence quenching was induced by red actinic light (635 nm) with various intensities. The reaction buffer contained 0.025% (w/v) DDM to achieve the trimeric form of LHCII. The light-induced change in absorbance of LHCII_3_ or PPBQ solution was detected using Cary 50 Bio spectrophotometer (Varian Inc., Australia) in the range from 250 to 800 nm.

### Confocal laser scanning microscopy (CLSM)

In *situ* experiments on LHCII_3_ migration to GE were performed with the use of the specially designated microscopic holder (Schematic diagram S2). CLSM images of the GE-LHCII_3_-PPBQ were made in a Nikon A1R MP inverted microscope equipped with galvo scanners and a Nikon S Plan Fluor ELWD 40 × Ph2 ADM lens. The final resolution of the image was set at 2048 × 2048 pixels (0.08 μm/pixel) in lateral dimensions and 1 μm in vertical dimensions. Fluorescence excitation was applied with a 488 nm diode laser, and emission was detected in the 570–620 nm wavelength range. Data stacks were deconvoluted using AutoQuant X3.0.5 software (Media Cybernetics Inc. MD, USA) and the three-dimensional fluorescence surface models were created using Imaris 8.4.0 software (Bitplane AG, Switzerland).

## Supplementary Information

Supplementary Information.

## References

[1] Rutherford AW, Osyczka A, Rappaport F (2012). Back-reactions, short-circuits, leaks and other energy wasteful reactions in biological electron transfer: Redox tuning to survive life in O(2). FEBS Lett..

[2] Liguori N, Periole X, Marrink SJ, Croce R (2015). From light-harvesting to photoprotection: Structural basis of the dynamic switch of the major antenna complex of plants (LHCII). Sci. Rep..

[3] Barros T, Kühlbrandt W (2009). Crystallisation, structure and function of plant light-harvesting Complex II. Biochem. Biophys. Acta..

[4] Robinson MT, Gizzie EA, Mwambutsa F, Cliffel DE, Jennings GK (2017). Mediated approaches to photosystem I-based biophotovoltaics. Curr. Opin. Electrochem..

[5] Zhang JZ, Reisner E (2020). Advancing photosystem II photoelectrochemistry for semi-artificial photosynthesis. Nat. Rev. Chem..

[6] Teodor AH, Bruce BD (2020). Putting photosystem I to work: Truly green energy: Trends in biotechnology. Trends Biotechnol..

[7] Brinkert K (1857). Photocurrents from photosystem II in a metal oxide hybrid system: Electron transfer pathways. Biochem. Biophys. Acta..

[8] Wang P (2021). Closing the gap for electronic short-circuiting: Photosystem I mixed monolayers enable improved anisotropic electron flow in biophotovoltaic devices. Angew. Chem. Int. Ed..

[9] Friebe VM (2016). Plasmon-enhanced photocurrent of photosynthetic pigment proteins on nanoporous silver. Adv. Func. Mater..

[10] Wolfe KD (2020). Photosystem I multilayers within porous indium tin oxide cathodes enhance mediated electron transfer. ChemElectroChem.

[11] Caterino R (2015). Photocurrent generation in diamond electrodes modified with reaction centers. ACS Appl. Mater. Interfaces..

[12] Piotrowska P (2019). Electrochemical characterization of LHCII on graphite electrodes–potential-dependent photoactivation and arrangement of complexes. Bioelectrochemistry.

[13] Izzo M (2021). Development of a novel nanoarchitecture of the robust photosystem I from a volcanic microalga cyanidioschyzon merolae on single layer graphene for improved photocurrent generation. Int. J. Mol. Sci..

[14] Morlock S, Subramanian SK, Zouni A, Lisdat F (2021). Scalable three-dimensional photobioelectrodes made of reduced graphene oxide combined with photosystem I. ACS Appl. Mater. Interfaces..

[15] Rochaix JD (1807). Regulation of photosynthetic electron transport. Biochem. Biophys. Acta..

[16] Fu HY (2017). Redesigning the QA binding site of photosystem II allows reduction of exogenous quinones. Nat. Commun..

[17] Shevela D, Messinger J (1817). Probing the turnover efficiency of photosystem II membrane fragments with different electron acceptors. Biochem. Biophys. Acta..

[18] Barbafina A (2008). Photophysical properties of quinones and their interaction with the photosynthetic reaction centre. Photochem. Photobiol. Sci..

[19] Friebe VM (1857). On the mechanism of ubiquinone mediated photocurrent generation by a reaction center based photocathode. Biochem. Biophys. Acta..

[20] Zhao F, Ruff A, Rögner M, Schuhmann W, Conzuelo F (2019). Extended operational lifetime of a photosystem-based bioelectrode. J. Am. Chem. Soc..

[21] Frigaard N-U, Tokita S, Matsuura K (1999). Exogenous quinones inhibit photosynthetic electron transfer in Chloroflexus aurantiacus by specific quenching of the excited bacteriochlorophyll c antenna. Biochem. Biophys. Acta..

[22] Lambrev PH (1807). Functional domain size in aggregates of light-harvesting complex II and thylakoid membranes. Biochem. Biophys. Acta..

[23] Müh F, Madjet MEA, Renger T (2010). Structure-based identification of energy sinks in plant light-harvesting complex II. J. Phys. Chem. B.

[24] Lambrev PH, Akhtar P, Tan HS (2020). Insights into the mechanisms and dynamics of energy transfer in plant light-harvesting complexes from two-dimensional electronic spectroscopy. Biochem. Biophys. Acta..

[25] Nagata M (2012). Immobilization and photocurrent activity of a light-harvesting antenna complex II, LHCII, isolated from a plant on electrodes. ACS Macro Lett..

[26] Yang Y (2014). Effect of the LHCII pigment-protein complex aggregation on photovoltaic properties of sensitized TiO2 solar cells. Phys. Chem. Chem. Phys..

[27] Yu D (2015). Enhanced photocurrent production by bio-dyes of photosynthetic macromolecules on designed TiO2 film. Sci. Rep..

[28] Lämmermann N (2019). Extremely robust photocurrent generation of titanium dioxide photoanodes bio-sensitized with recombinant microalgal light-harvesting proteins. Sci. Rep..

[29] Li F (2020). Electrostatic adsorption of a fluorophores-modified light-harvesting complex II on TiO2 photoanodes enhances photovoltaic performance. J. Power Sources.

[30] Compton, R. G. & Banks, C. E. *Understanding Voltammetry, 3rd edition*. (World Scientific Publishing Europe, 2018).

[31] Huynh MT, Colin WA, Cavell AC, Stahl SS, Hammes-Schiffer S (2016). Quinone 1 e– and 2 e–/2 H+ reduction potentials: Identification and analysis of deviations from systematic scaling relationships. J. Am. Chem. Soc..

[32] Zubik M (1827). The negative feedback molecular mechanism which regulates excitation level in the plant photosynthetic complex LHCII: Towards identification of the energy dissipative state. Biochem. Biophys. Acta..

[33] Karsili TNV, Tuna D, Ehrmaier J, Domcke W (2015). Photoinduced water splitting via benzoquinone and semiquinone sensitisation. Phys. Chem. Chem. Phys..

[34] Mennenga A, Gartner W, Lubitz W, Görner H (2006). Effects of noncovalently bound quinones on the ground and triplet states of zinc chlorins in solution and bound to de novo synthesized peptides. Phys. Chem. Chem. Phys..

[35] Razeghifard MR, Wydrzynski T (2003). Binding of Zn-chlorin to a synthetic four-helix bundle peptide through histidine ligation. Biochemistry.

[36] Razeghifard R (2015). Photochemistry of free and bound Zn-chlorophyll analogues to synthetic peptides depend on the quinone and pH. J. Photochem. Photobiol. B, Biol..

[37] Ostroumov EE, Götze JP, Reus M, Lambrev PH, Holzwarth AR (2020). Characterization of fluorescent chlorophyll charge-transfer states as intermediates in the excited state quenching of light-harvesting complex II. Photosynth. Res..

[38] Lokstein H, Renger G, Götze JP (2021). Photosynthetic light-harvesting (antenna) complexes-structures and functions. Molecules.

[39] De Causmaecker S, Douglass JS, Fantuzzi A, Nitschke W, Rutherford AW (2019). Energetics of the exchangeable quinone, Q_B_, in photosystem II. Proc. Natl. Acad. Sci. USA.

[40] Szewczyk S, Białek R, Burdziński G, Gibasiewicz K (2020). Photovoltaic activity of electrodes based on intact photosystem I electrodeposited on bare conducting glass. Photosynth. Res..

[41] Ciesielski PN (2010). Enhanced photocurrent production by photosystem I multilayer assemblies. Adv. Func. Mater..

[42] Kirchhoff H, Hinz HJ, Rosgen J (2003). Aggregation and fluorescence quenching of chlorophyll a of the light-harvesting complex II from spinach in vitro. Biochem. Biophys. Acta..

[43] Gruszecki WI (2009). Light-induced change of configuration of the LHCII-bound xanthophyll (tentatively assigned to violaxanthin): A resonance Raman study. J. Phys. Chem. B.

[44] Palinska A (2010). Methylene blue interactions with chromosomal and plasmid DNA on screen-printed carbon electrodes. Electroanalysis.

[45] Shukla MK (2020). A novel method produces native light-harvesting complex II aggregates from the photosynthetic membrane revealing their role in nonphotochemical quenching. J. Biol. Chem..

