# Supplementary material for: The coupled photocycle of phenyl-p-benzoquinone and Light-Harvesting Complex II (LHCII) within the biohybrid system
Source: Sci Rep. 2022 Jul 27;12:12771. doi: 10.1038/s41598-022-16892-y (PMC9329374; doi:10.1038/s41598-022-16892-y)
Supplement: Supplementary file 1 — Supplementary Information. [file 41598_2022_16892_MOESM1_ESM.docx]

**Supplementary Information**

**The coupled photocycle of phenyl-p-benzoquinone and Light Harvesting Complex II (LHCII) inside the biohybrid system**

Magdalena Łazicka^1^, Adriana Palińska-Saadi^2, 3^, Paulina Piotrowska^1^, Bohdan Paterczyk^4^, Radosław Mazur^1^, Magdalena Maj-Żurawska^2^, Maciej Garstka^1^

^1^ Department of Metabolic Regulation, Institute of Biochemistry, Faculty of Biology, University of Warsaw, Miecznikowa 1, 02-096 Warsaw, Poland

^2^ Laboratory of Basics of Analytical Chemistry, Faculty of Chemistry, University of Warsaw, Pasteura 1, 02-093 Warsaw, Poland

^3^ Bioanalytical Laboratory, Biological and Chemical Research Centre, University of Warsaw, Żwirki i Wigury 101, 02-089 Warsaw, Poland

^4^ Laboratory of Electron and Confocal Microscopy, Faculty of Biology, University of Warsaw, Miecznikowa 1, 02-096 Warsaw, Poland

*Corresponding author: Maciej Garstka, phone: 48 22 5543213, e-mail: m.garstka@uw.edu.pl: ORCID: 000-0001-7141-505X


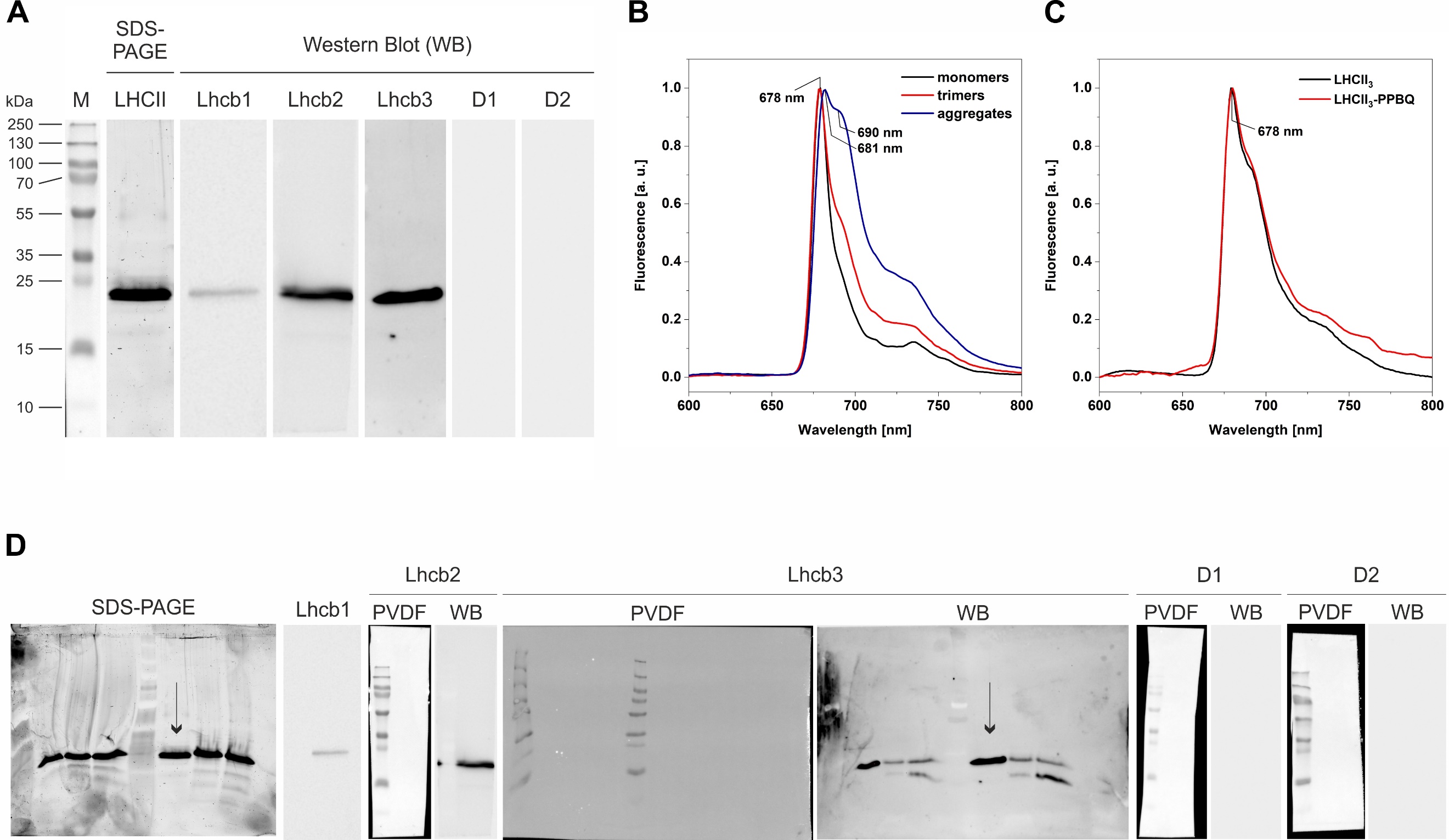


**Supplemental Figure S1**

Characterization of LHCII preparation from spinach.

(**A**) SDS-PAGE of LHCII stained with Sypro Ruby and immuno-detection analysis of antenna (Lhcb1, Lhcb2, Lhcb3) and core (D1, D2) proteins. (**B**) Fluorescence emission spectra at 77K (excitation at 440 nm) of LHCII in the monomeric (black), trimeric (red), and aggregates (blue line) form. (**C**) Fluorescence spectra of LHCII at 77K in the trimeric form in the presence (red) or absence (black) of 0.1 mM PPBQ. Panel **D** shows the original SDS-PAGE, PVDF membranes and Western blots (WB) presented in panel A.

LHCII proteins were detected by the SDS-PAGE using 12% polyacrylamide gel. The gel was stained with Sypro Ruby as previously described ^1^. The Sypro Ruby fluorescence was detected using the ChemiDoc Imaging System (Bio-Rad). The Western blots were performed as described in ^2^ using primary antibodies Lhcb1 (AS01 004), Lhcb2 (AS01 003), Lhcb3 (AS01 002), D1 (AS10 704), D2 (AS06 146) (Agrisera, Sweden). The Western blot signals were visualized using luminol and coumaric acid (buffer: 0.197 mM coumaric acid, 1.562 mM luminol in 100 mM Tris pH 8.5 containing 0.009% (v/v) H_2_O_2_). After 2 minutes in darkness, the signal was detected using the ChemiDoc Imaging System (Bio-Rad).

Fluorescence emission spectra of LHCII at low temperature (77 K) were recorded using the modified Shimadzu RF-5301PC spectrofluorimeter. Oligomeric states were induced using different concentrations of n-Dodecyl-β-D-maltoside (DM) or MgCl_2_ (samples contained: monomers with 1% DM, trimers with 0.025% DM, aggregates with 40 mM MgCl_2_). Samples were placed in a polytetrafluoroethylene cuvette and frozen in liquid nitrogen. All spectra were recorded through the LP600 filter. The LHCII samples were diluted to 10 µg Chl/ml using HEPES buffer without or with 0.1 mM PPBQ. The chlorophyll concentration was measured as described previously ^3^. The spectra were normalized to the unity at maxima of fluorescence. The emission spectrum of LHCII monomers and trimers showed a maximum at 678 nm, whereas the maximum for aggregated form was shifted to 682 nm. The different degree of aggregation between samples was confirmed by F735/F680 ratio ^4,5^, indicating that preparation in presence of 0.025% (w/v) DDM consists mainly of trimeric form of LHCII (LHCII_3_).


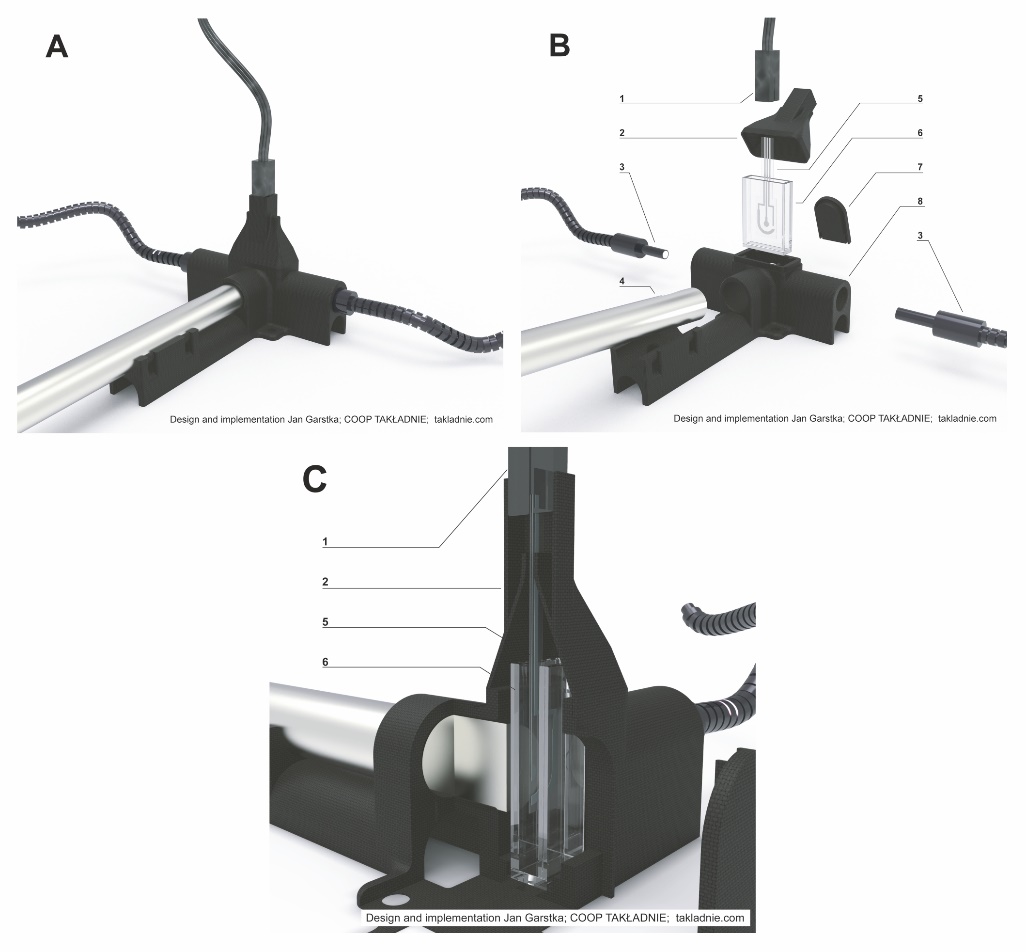


**Schematic diagram S1**

The special holder for simultaneous detection of fluorescence emission and photocurrent generation.

Overall view of holder (**A**), diagram of holder with component breakdown (**B**), cross section of holder (**C**). The electrode clamp, **1**; upper cover of holder, **2**; actinic light source by fiber optics, **3**; quartz tube connected to the fiber optics from the spectrofluorimeter, **4**; graphite electrode, **5**; glass cuvette, **6**; back cover of holder, **7**; main body of holder, **8**. © Jan Garstka, COOP TAKŁADNIE, takladnie.com


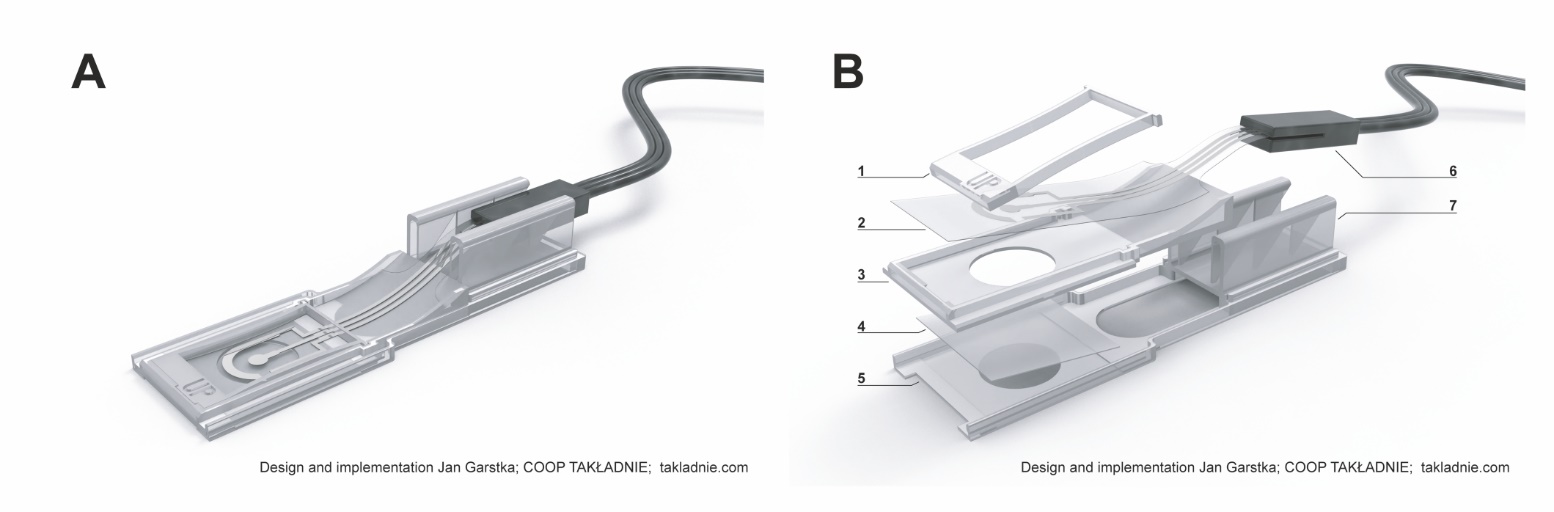


**Schematic diagram S2**

The holder for *in situ* microscopic imaging of electrode surface.

Overall view of holder (**A**), diagram of holder with component breakdown (**B**). The upper clamp, **1**; graphite electrode, **2**; container with electrode buffer, **3;** microscopic coverslip, **4**; bottom clamp, **5**; electrode clamp, **6**; stabilizer for electrode clamp, **7**. © Jan Garstka, COOP TAKŁADNIE, takladnie.com


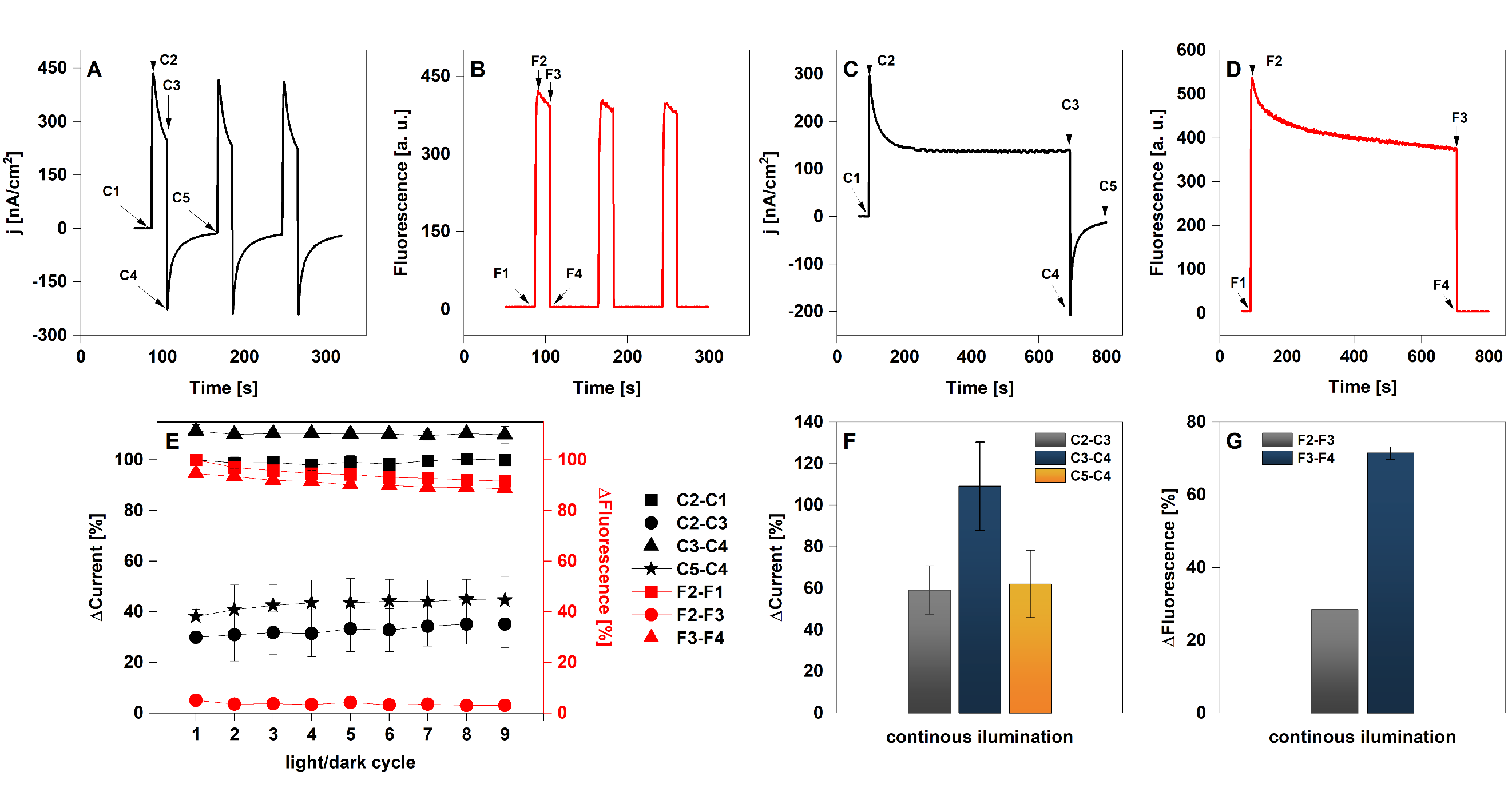


**Supplemental Figure S2**

Statistical analyses of changes of Chl fluorescence and photocurrent intensity.

Fluorescence and photocurrent intensity were recorded at the same time during the dark/light cycle (**A**, **B**, **E**) and continuous illumination (**C**, **D**, **F**). The blue light as an actinic light was used. Figures **A** and **B** show which points were taken into account to calculate the intensity of a particular signal. The data presented in figures **E**, **F**, **G** were calculated as a percentage of the first maximal value estimated for photocurrent or fluorescence (C2-C1 or F2-F1). The data are mean values ± SD for 4 independent experiments.

The fluorescence quenching effects in the range of 3-4% and 28% were observed for dark/light cycles and for continuous illumination, respectively (Fig. S2E, G). Similarly, the photocurrent level decreased by about 30% and 60% for dark/light cycles and continuous illumination (Fig. S2E, F), respectively. Nevertheless, the maxima of anodic photocurrent were invariable under the nine dark/light cycles (Fig. S2E), and, except for the 60 s declining period, the anodic current flow was stable under the continuous illumination (Fig. S2C, F). The cathodic dark-current achieved the values equal 45% and 60% of the initial value of anodic photocurrent within second (Fig. S2E, F), for both types of experimental conditions, respectively.

The observed photocurrent quenching is probably related to transient processes in the diffusion layer on the GE surface, what is similar to current decay resulting from the potential step in amperometry ^6^. On the other hand, the dark-current generation can depend on the equilibrium between redox forms of PPBQ. Turning off the illumination induces the short-time cathodic current due to the reduction of accumulated oxidized forms of PPBQ, followed by its decrease and return to the equilibrium state that was found before illumination.


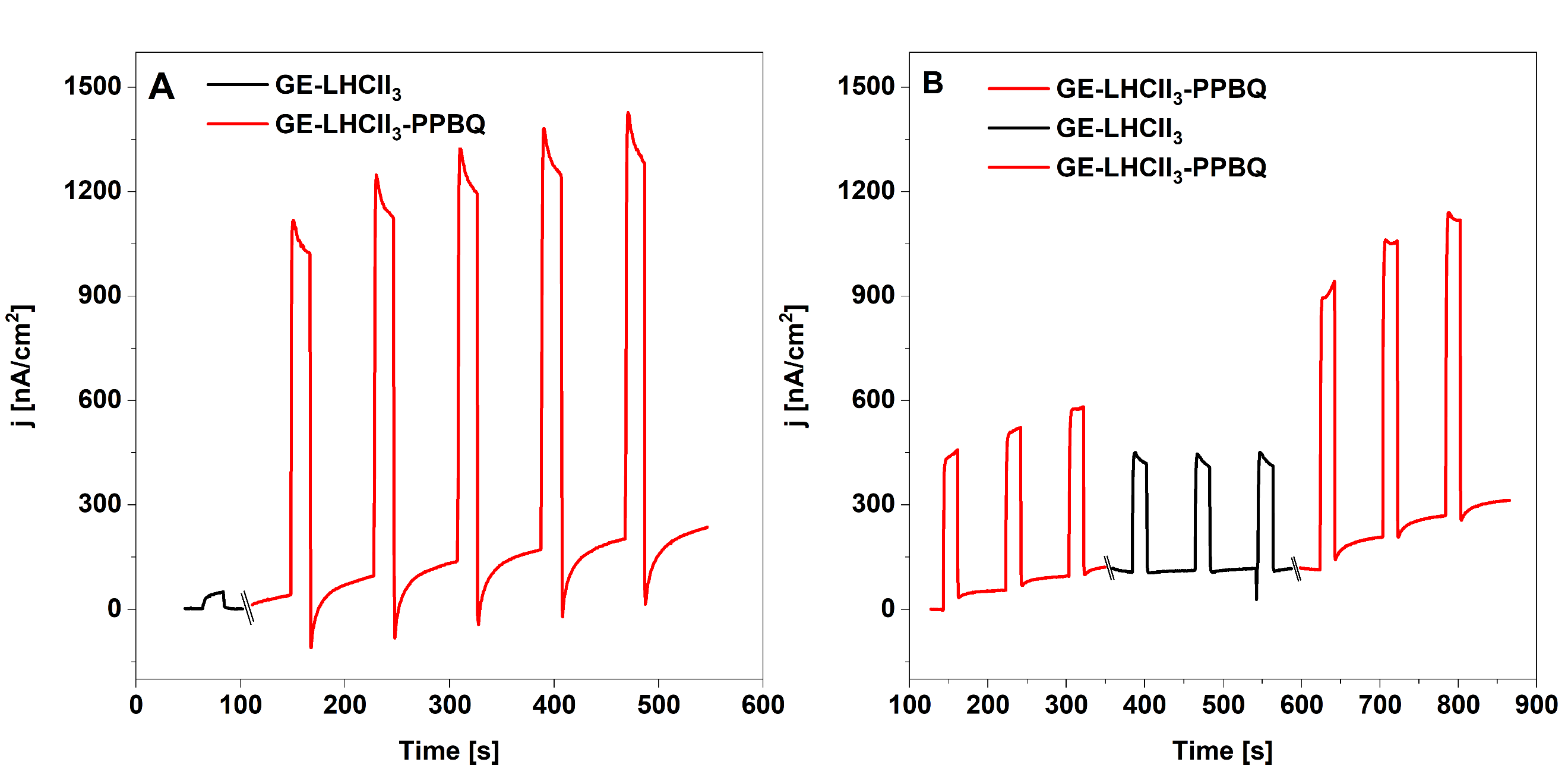


**Supplemental Figure S3**

Influence of PPBQ on photocurrent generation.

Photocurrent responses to illumination of the GE-LHCII_3_ system with actinic white light (4900 μE) in HEPES buffer in the absence of PPBQ (black line) and after adding the PPBQ (red line) to the final concentration of 0.1 mM (A) and after replacement of the 0.1 mM PPBQ solution for buffer containing no mediator (dark line) and subsequent exchange to buffer with 0.1 mM PPBQ (red line) (**B**).

Data presented in Fig. S3 and in Figs. 1 and 2 indicate that the interaction between LHCII and PPBQ is a critical process for light-dependent current generation and that the limiting factor of efficiency of the GE-LHCII_3_-PPBQ system is not light harvesting, but energy transfer from LHCII_3_ to GE mediated by PPBQ.


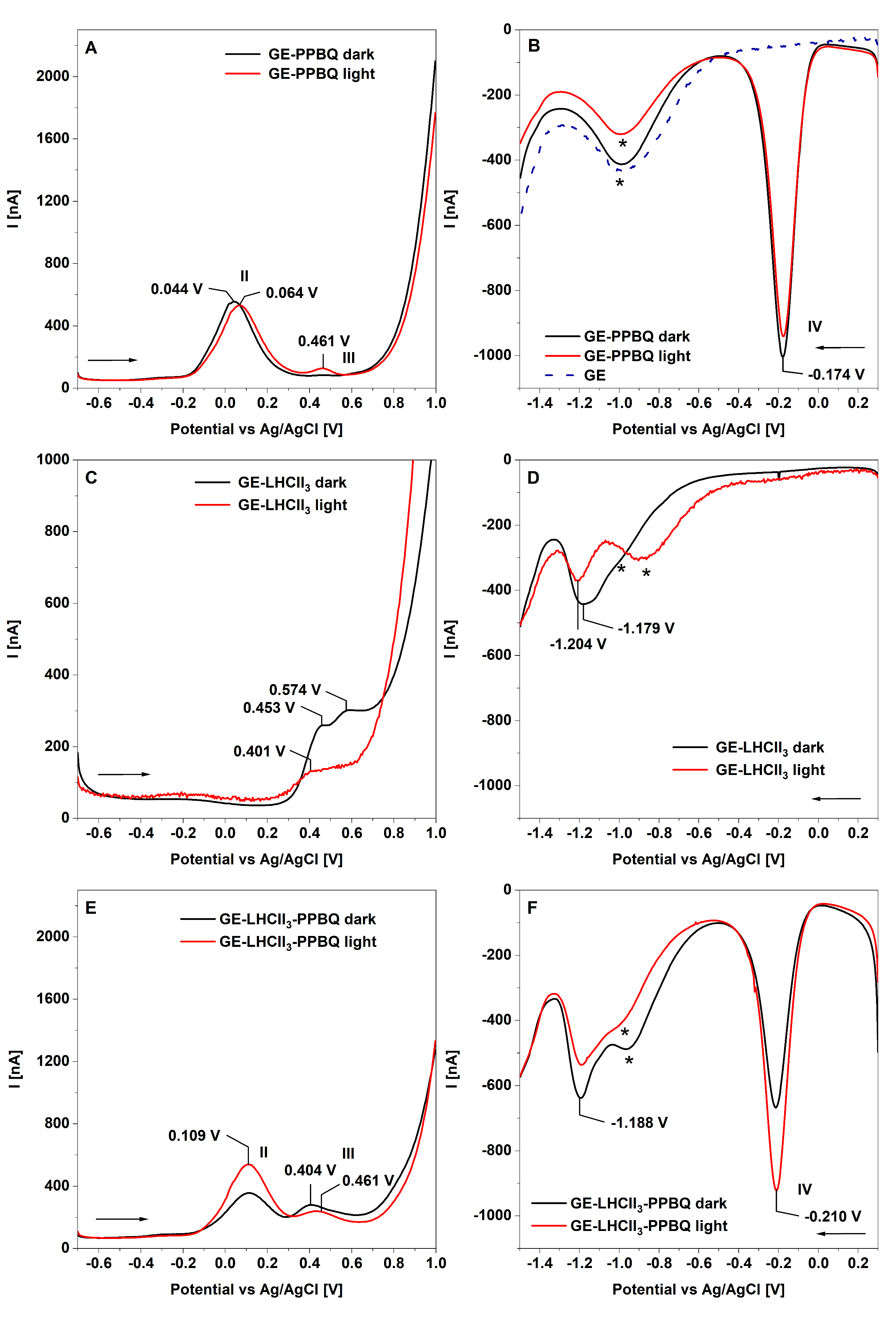


**Supplemental Figure S4**

Voltammetric characterization of PPBQ and LHCII_3_ under dark and light treatments.

The differential pulse voltammograms (DPV) of GE-PPBQ (**A**, **B**), GE-LHCII_3_ (**C**, **D**) and GE-LHCII_3_-PPBQ (**E**, **F**) monitoring oxidation (**A**, **C**, **E**) and reduction (**B**, **D**, **F**) processes were recorded in the dark (black lines) and light (red lines) conditions. The measurements were carried out in special holders and samples were illuminated with actinic white light with the intensity of 4874 µE. The arrows indicate the direction of potential change. The asterisks indicate the current minima observed for the bare GE (blue line in B). Curves were smoothed using a polynomial fixed order available in NOVA software. The presented voltammograms are representative of at least 3 separate experiments.

The differential pulse voltammetry (DPV) in the wide potential scan range for the GE-PPBQ system revealed great oxidation and reduction peaks at roughly +0.055 V (peak II) and −0.180 V (peak IV) vs. Ag/AgCl, respectively (Fig. S4A, B). The signals for LHCII_3_ deposited on GE were significantly far away from peaks for PPBQ and detected in the range from +0.450 to +0.570 V and around −1.200 V for oxidation and reduction, respectively (Fig. S4C, D). The range of voltammetric values for LHCII_3_ was in line with data for other CP complexes ^7^ and was derived from the electrochemical properties of Chl molecules ^8,9^. The DPV scans of the GE-LHCII_3_-PPBQ system revealed that the reduction scan was a simple composition of scans for GE-PPBQ and GE-LHCII_3_ with peaks around −0.210 V (peak IV) and –1.200 V (Fig. S4F). However, the oxidation scan revealed significant differences in respect to simpler systems (Fig. S4E versus S4A).


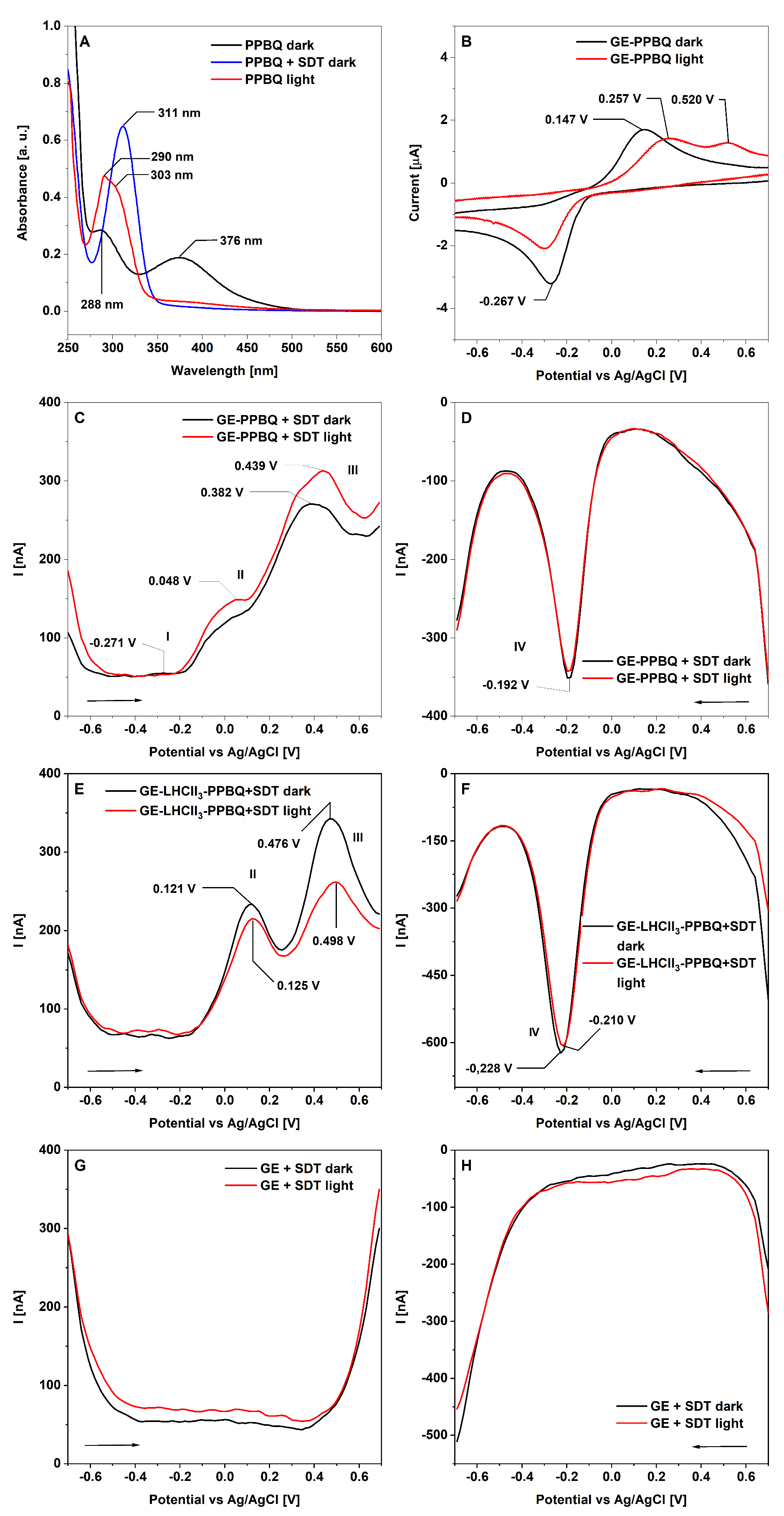


**Supplemental Figure S5**

Effect of light and sodium dithionite (SDT) reduction on absorbance spectra and voltammograms of PPBQ and LHCII_3_ under dark and light conditions.

Absorbance spectra of 0.1 mM PPBQ solution (**A**) measured in dark (black line) and after 10 min of illumination (red line) or its reduction with 0.1 mM SDT under dark condition (blue line). Cyclic voltammetry (CV) of GE-PPBQ under dark (black line) and light (red line) conditions (**B**). Differential pulse voltammograms (DPV) of GE-PPBQ (**C**, **D**), GE-LHCII_3_-PPBQ (**E**, **F**), and GE (**G**, **H**) under dark (black lines) and light (red lines) conditions in the presence of 0.1 mM SDT. The arrows indicate the direction of the potential change. The measurements were performed as described in the caption to Fig. S4. The presented data are representative of at least 3 separate experiments.

The absorption spectra of the PPBQ solution recorded under dark conditions revealed the two maxima at 288 and 376 nm (black line in Fig. S5A) corresponding to the reduced and oxidized forms of quinones, respectively. After 10 minutes of illumination of the sample, the band related to the oxidized form of PPBQ completely disappeared and a new band composed of two overlapping bands at 290 and 303 nm was noticed (red line in Fig. S5A). These absorption changes were correlated with the light-induced appearance of anodic peak III (Fig. 4A, S6A, Tab. S1). Summarizing the voltammetric analysis of the PPBQ solution, it can be stated that the potentials of three peaks (II, III, and IV) change after illumination with actinic light (Tab. S1), which would indicate the appearance of new, probably radical forms of this compound ^10-12^.

The 290-303 nm absorption band partially overlapped with the spectrum centered at 311 nm obtained for chemically reduced PPBQ by sodium dithionite (SDT) (blue line in Fig. S5A). Furthermore, the DPV voltammograms of PPBQ reduced with SDT under the dark conditions revealed a dominated peak III with a maximum at +0.382 V, evidently composed of overlapping peaks (black line in Fig. S5C). The DPV scan under the light conditions revealed a similar composed character of peak III with the maximum shifted to +0.439 V (red line in Fig. S5C). The shape of the chemically induced peaks III is different from the light-induced ones (Fig. 4A, Tab. S1), suggesting either that we are dealing with various forms of PPBQ or that only one of the SDT-induced forms is the same as the form induced by light. The other SDT-induced peaks, the IV around −0.192 V, the shoulder of III (+0.439 V), and a trace of I (Fig. S5C, D) were correlated with light-induced peaks of PPBQ (Tab. S1). Furthermore, the characteristic for the GE-LHCII_3_-PPBQ peak II was also detected in the presence of SDT, that is, in the reducing environment (Fig. S5E).


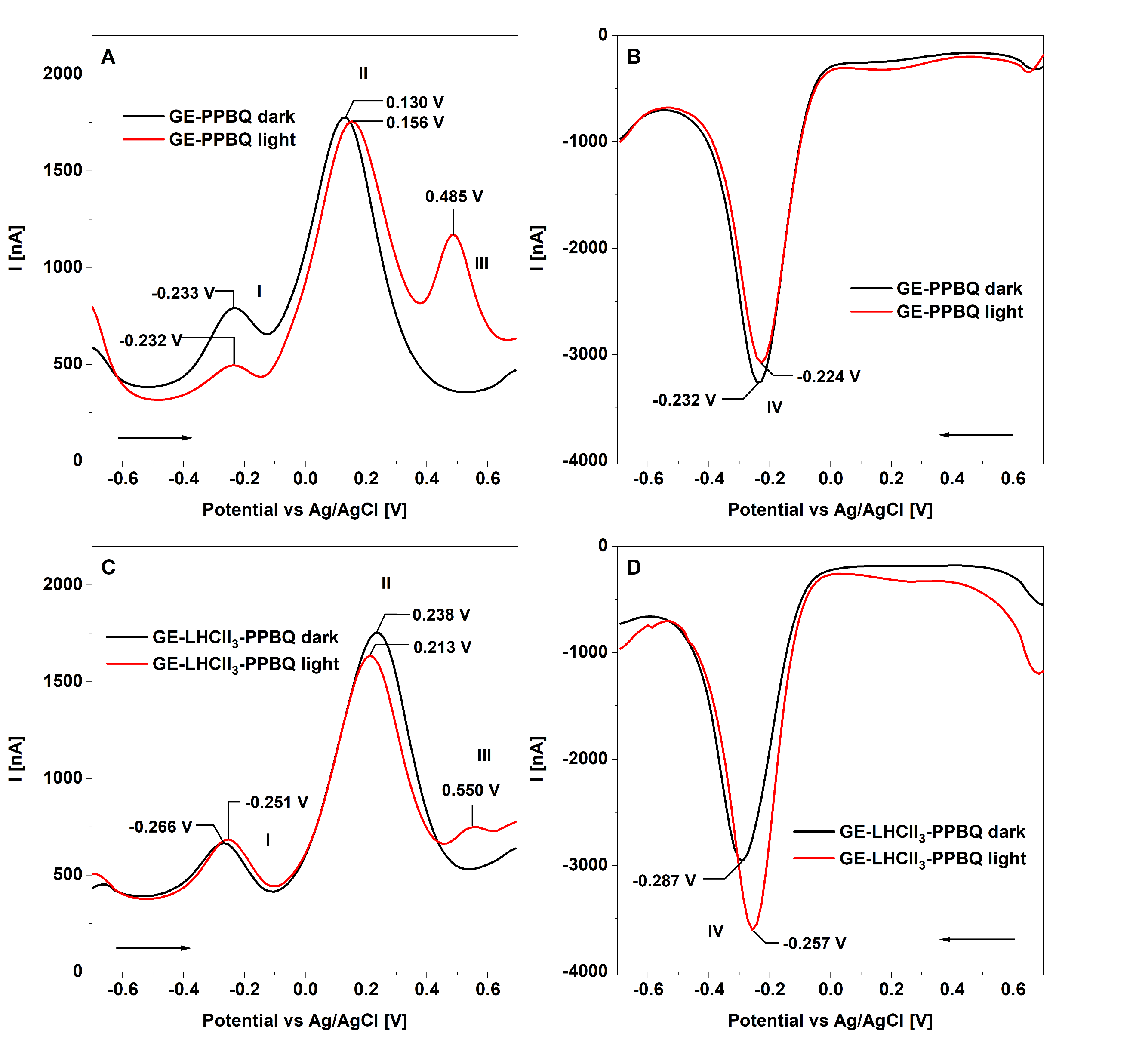


**Supplemental Figure S6**

Voltammetric characterization of the interaction between LHCII_3_ and PPBQ.

Square-wave voltammograms (SWV) of the oxidation (**A**, **C**) and reduction (**B**, **D**) processes of GE-PPBQ (**A**, **B**) and GE-LHCII_3_-PPBQ (**C**, **D**) under dark (black lines) and light (red lines) conditions. The arrows indicate the direction of the potential change. The measurements were performed as described in the caption to Fig. S4. The presented voltammograms are representative of at least 3 separate experiments.

**
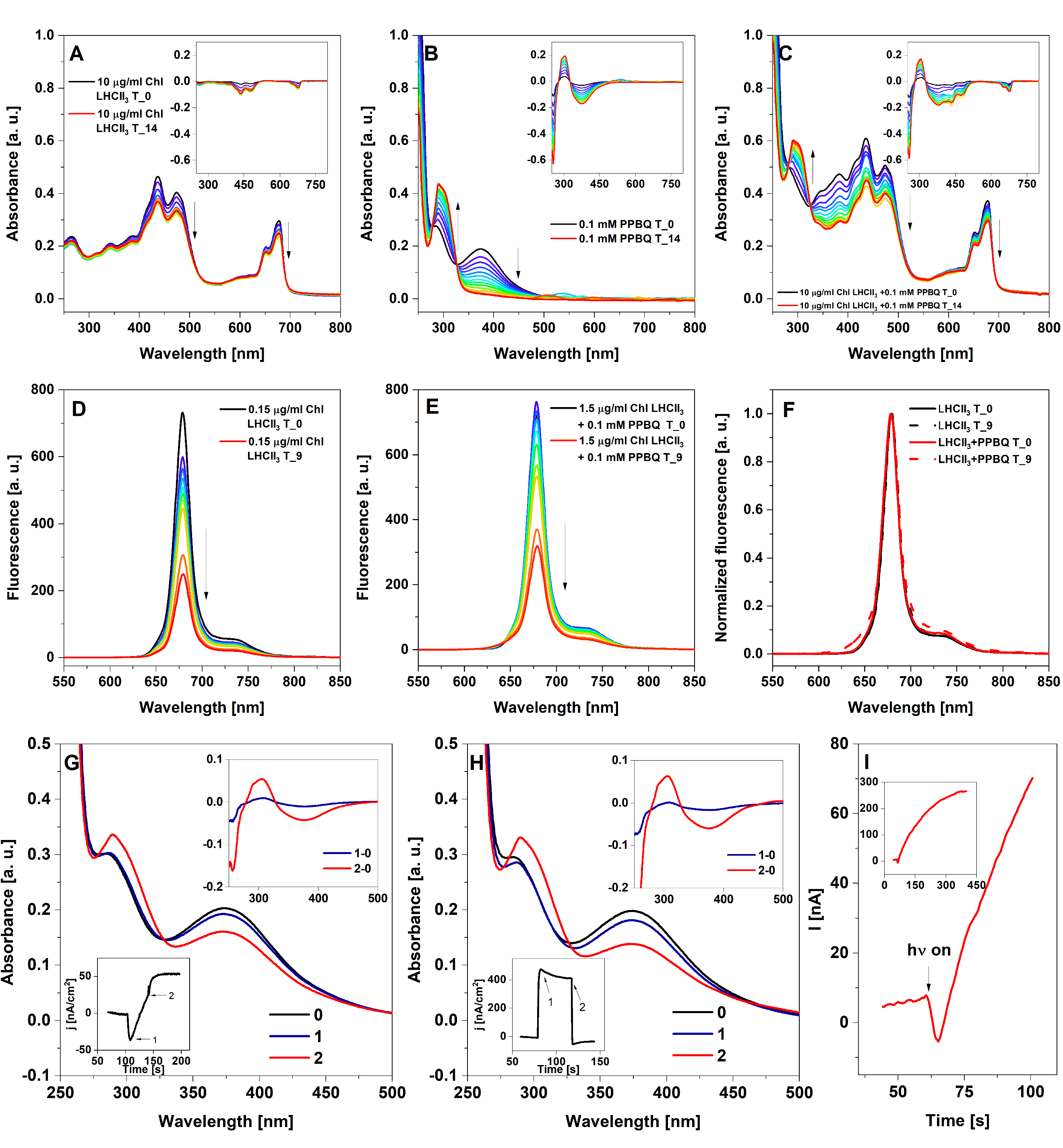
**

**Supplemental Figure S7**.

Illumination effects on the absorption and fluorescence spectra of LHCII_3_ and PPBQ in solution and buffer phase of electrochemical systems.

Absorption spectra of LHCII_3_ (10 Chl µg/ml) (**A**), PPBQ (0.1 mM) (**B**), and LHCII_3_ + PPBQ (**C**) were recorded after successive 20 s sample illumination periods. The black lines indicate the spectra before illumination, the lines depicted with the blue, green, and red colors indicate spectra after successive illumination. The insets present the difference spectra, i.e. the dark spectrum minus consecutive light spectra. Room-temperature fluorescence spectra of solution of LHCII_3_ (0.15 Chl µg/ml) (**D**) and LHCII_3_ + PPBQ (1.5 Chl µg/ml). Normalized fluorescence for initial and final spectra of LHCII_3_ and LHCII_3_ + PPBQ samples (**E**).

The PPBQ spectra were recorded in the buffer phase of the GE-PPBQ (**G**) and GE-LHCII_3_-PPBQ (**H**) systems under chronoamperometric measurements. The black line indicates the initial spectra, and the blue and red lines indicate spectra recorded in buffer withdrawn from the reaction cuvette at the time points depicted in the insets in the bottom left corner. The difference spectra are presented in the insets on the upper right corner. The photocurrent was generated in the GE-PPBQ system under long-time illumination (**I**), and the inset shows the whole course of the measurement. The samples were illuminated with actinic white light with an intensity of 4900 µE. The presented data are representative of 3 separate experiments.

The cyclic illumination of the LHCII_3_ solution caused a gradual decrease of the overall absorbance by about 20% (Fig. S7A). The cycle of twelve illuminations induces the reduction of PPBQ to forms revealed the overlapping absorbance maxima at 290 and 303 nm (Fig. S7B). The changes in absorbance of a mixture of LHCII_3_ and PPBQ measured after cyclic illuminations (Fig. 7C) were a simple composition of individual spectra, indicating that light-induced reduction of PPBQ occurred independently on the presence of LHCII_3_. Furthermore, the effect of PPBQ on changes of LHCII_3_ absorbance was unnoticeable (Fig. S7C versus S7A), although PPBQ is an efficient fluorescence quencher (Fig. 3).

The initial Chl fluorescence level of LHCII_3_ at a concentration of 0.15 Chl µg/ml (Fig. S7D) was the same as the initial Chl fluorescence in the presence of PPBQ but for a LHCII_3_ sample ten times more concentrated (Fig. S7E). Cyclic illumination of both samples resulted in a gradual decrease of fluorescence, but given the differences in sample concentrations, these changes were higher for the LHCII_3_ solution (Fig. S7E). This indicates that PPBQ quenches the fluorescence already at the starting point of the measurement. Analysis of LHCII_3_ + PPBQ normalized fluorescence spectra (Fig. S7F), according to Lingvay et al. ^13^, revealed that slight shoulder at 650 may be related to photobleaching of Chl molecules.

Analysis of light-induced changes in absorbance and fluorescence spectra of LHCII_3_ solutions (Fig. S7A, C, Fig. S7D, E) indicates that the observed quenching process might be related to the aggregation of LHCII ^14^. However, the partial photobleaching of the Chl molecules in presence of PPBQ was observed ^13^. These observations confirmed also that PPBQ is efficient Chl quencher, and the total fluorescence quenching is the resultant of the light and PPBQ effects (Fig. 3)

The photoreduction of PPBQ was also analyzed concerning photocurrent generation in the GE-PPBQ and GE-LHCII_3_-PPBQ systems. In both cases, identical changes of absorbance at 290 nm were detected in the buffer phase, indicating the relation of the reduction of PPBQ to the illumination time (Fig. 7G, H), but not to the photocurrent generation, which in the GE-PPBQ system revealed the alternating flow, whereas in the GE-LHCII_3_-PPBQ system it has only the anodic direction. These observations suggest that in the buffer phase an accumulation of reduced forms of PPBQ occurred independently on electrochemical reactions running on the GE surface. Furthermore, the generation of photocurrent did not result in the release of LHCII_3_ into the buffer phase, as shown in the absorption spectra (Fig. S7H).

The PPBQ-induced cathodic photocurrent changed direction after 5 s (Fig. S7I), but the anodic photocurrent did not reach full saturation within 400 s (Fig. 5I, inset). This phenomenon might be explained by the presence of the oxidized forms of PPBQ at the GE surface, drawing electrons from GE, at the beginning of the illumination. Over time, the light-reduced forms of PPBQ appeared at the GE surface and the anodic current flow started.


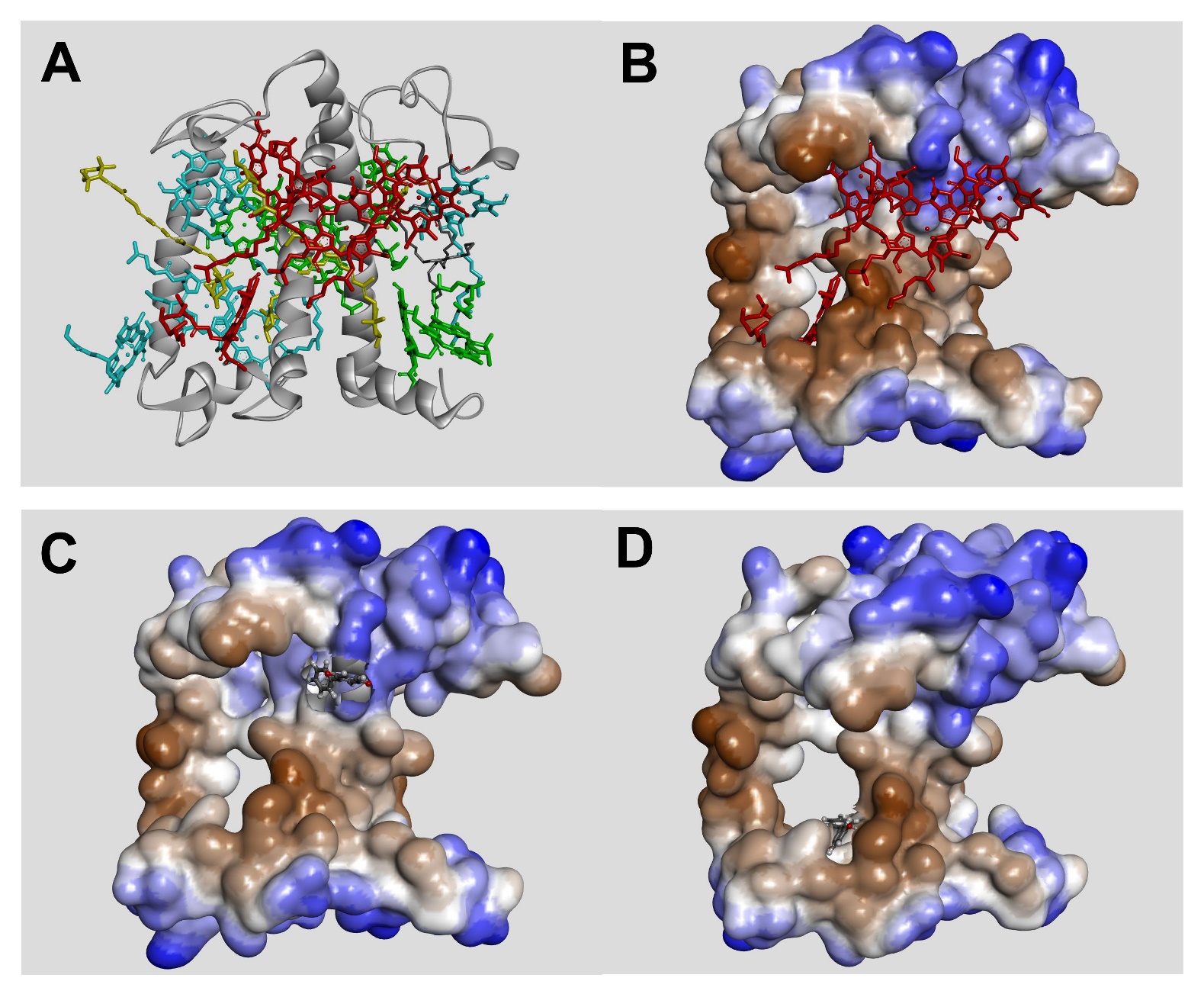


**Supplemental Figure S8**

Three-dimensional computing models of PPBQ binding to the Lhcb1 protein. The model of a monomer of LHCII imaging the binding sites of pigments within the protein (**A**), localization of 610/611/612 and 604 of Chl *a* molecules concerning protein hydrophobic surface (**B**), plausible docking site of PPBQ at stromal (**C**) and lumenal (**D**) parts of Lhcb1 protein.

In LHCII terminal emitter domain includes a cluster of three Chl *a* (610, 612, 611). Except for these coupled chlorophylls, Chl *a* 604 might also play the role of an alternative energy sink ^15,16^. Molecules 602, 603, 609, 613, 614 of Chl *a* and molecules 601, 605, 606, 607, 608 of Chl *b* are indicated in green and cyan, respectively. The 610/611/612 and 604 of Chl molecules are colored red. The xanthophylls are imaging in yellow. Hydrophobic regions are colored brown, whereas the more polar regions of the protein are depicted in blue. The Chl molecule numbers were named with commonly accepted nomenclature ^16,17^.

The models presented in Fig. S8C and D were depicted with the use of the Ligand Docking tool of the Rosetta Online Server That Includes Everyone (ROSIE) (https://rosie.graylab.jhu.edu/ligand_docking) ^18^ and spinach crystal structure of LHCII ^19^ (PDB entry 4LCZ). On the basis of the minimization of energies between the ligand and target structure, the ROSIE software predicts the plausible docking site of ligand ^20^. The final images were presented with the use of BIOVIA Discovery Studio 2020.

As was shown in the model (Fig. S8A), the cluster of 610/611/612 Chl *a* is located in the stromal layer of LHCII, at the boundary between the hydrophobic and hydrophilic domains of LHCII (Fig. S8B). The Chl *a* 604 is localized at the lumenal layer of LHCII (Fig. S8A), more immersed in the hydrophobic domain of the complex (Fig. S8B). All these Chls are situated on the outward part of the LHCII trimer. Computed by ROSIE software, the plausible docking sites of PPBQ in stromal as well as in lumenal regions of the protein facing the outside of the LHCII trimer have coincided with the binding sites for Chl *a* 612 (Fig. S8C) and Chl *a* 604 (Fig. S8D). These models do not consider the impacts of PPBQ with Chl molecules or LHCII-associated lipids ^3,21^, therefore the exact location of PPBQ in the LHCII requires further and more accurate computer simulations.


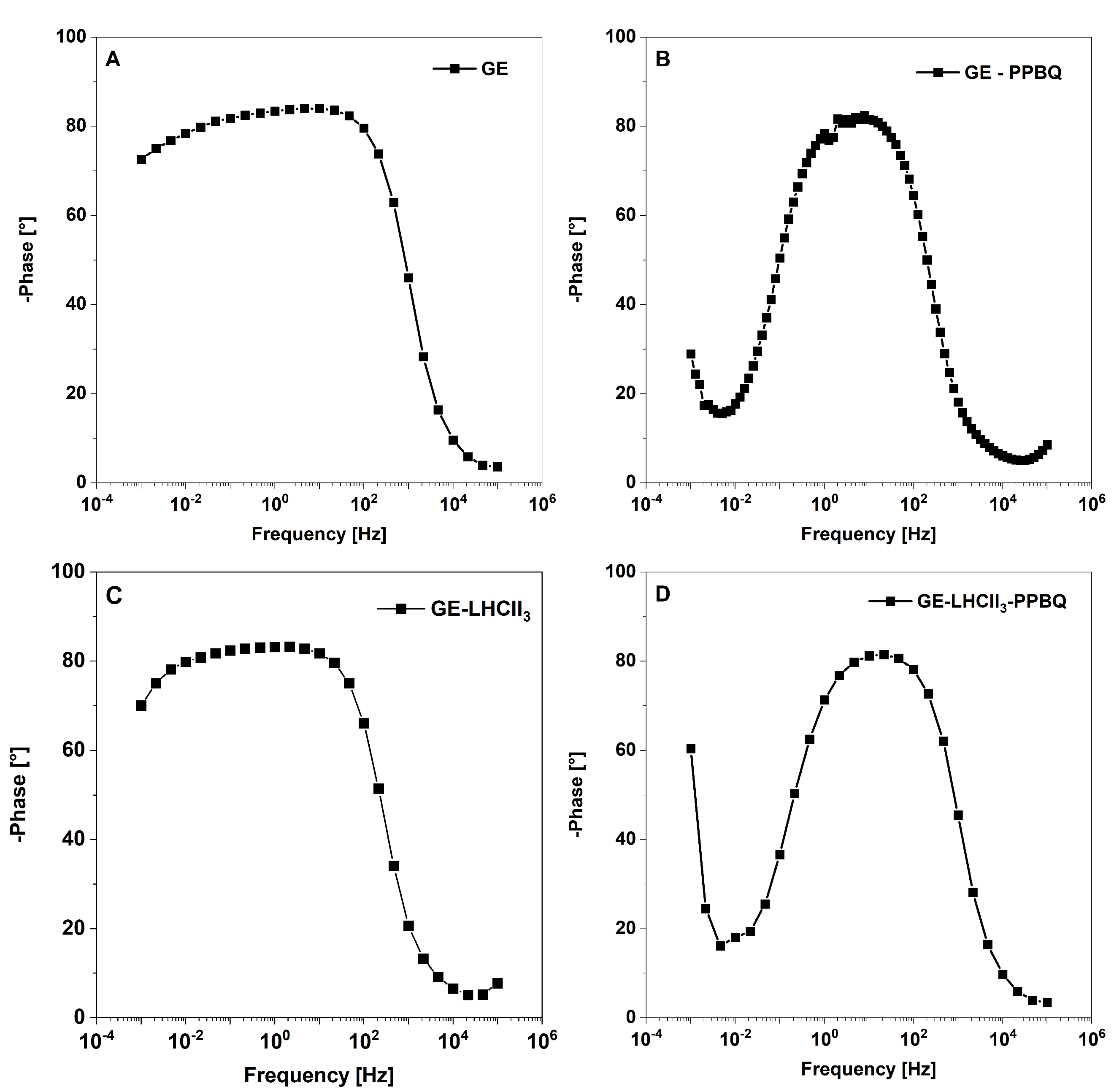


**Supplemental Figure S9**

Electrochemical impedance spectroscopy (EIS) spectra recorded for each modification step of the graphite electrode by scanning from 10^5^ to 10^–3^ Hz under dark conditions at OCP.

Bode phase plots of: unmodified GE electrode in HEPES buffer (**A**), GE in the presence of 0.1 mM mM PPBQ, GE-PPBQ (**B**), GE modified by 1.5 µl suspension of LHCII_3_ in glutaraldehyde matrix (0.2 µg Chl/µl), LHCII_3_-GE (**C**) and final biohybrid system GE-LHCII_3_-PPBQ (**D**). The plots are representative of 3 separate experiments.

Appearing the electrical double layer on the surface of solid electrode might be characterized by EIS. Using EIS, determination of a double-layer capacitance or a charge transfer resistance in the system with an unmodified electrode or in the presence of a modifier is possible. Among others, EIS enables describing quantitatively and qualitatively an electrochemical modification of electrodes resulting from binding of proteins, composed protein complexes, or charged molecules. EIS results are often represented in the form of Nyquist plots, which are usually composed of a semicircle part describing a charge transfer kinetics, and a linear part pointing to the diffusion processes that occur at lower frequencies. The other EIS presentation, the Bode plots, present the impedance and phase angle plotted against frequency, which makes it possible to determine the capacitative or inductive effects of electrochemical systems ^22^.


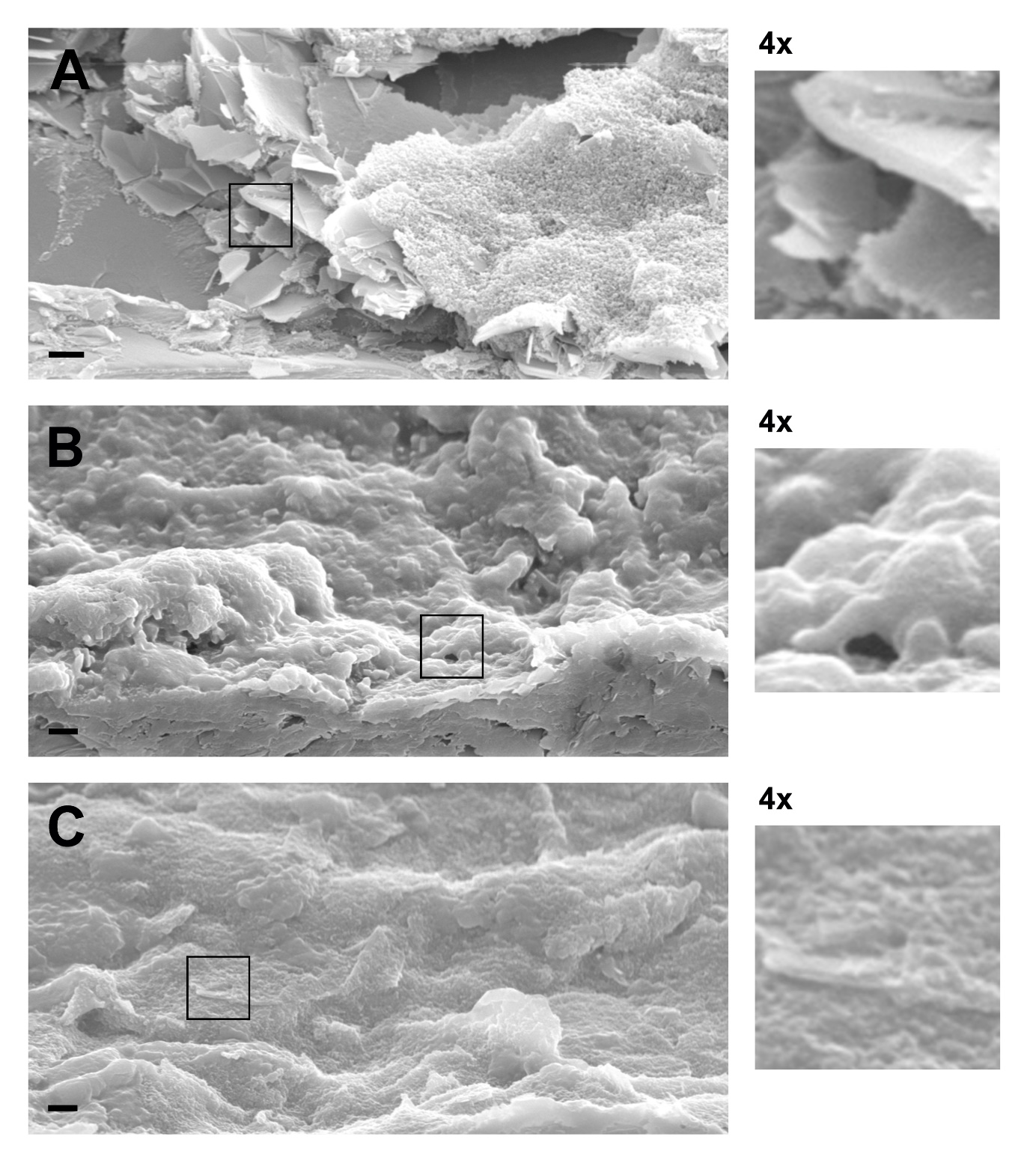


**Supplemental Figure S10**

Scanning electron microscopy (SEM) imaging of structures of LHCII_3_-GE register at a 30° angle.

The control graphite electrode layer (GE) (**A**), GE-LHCII_3_ layer before electrochemical treatment (**B**), GE-LHCII_3_ layer after 20 min treatments with open circuit potential (OCP) under dark conditions (**C**). Right panels are 4-times magnification of selected micrograph fragments. The left bottom bars are equal to 2 μm, whereas the microscopic magnifications are 5K (**A**) and 4K (**B**, **C**). SEM measurements were performed according to the protocol described previously ^3^. The images are representative of at least 5 independent experiments.


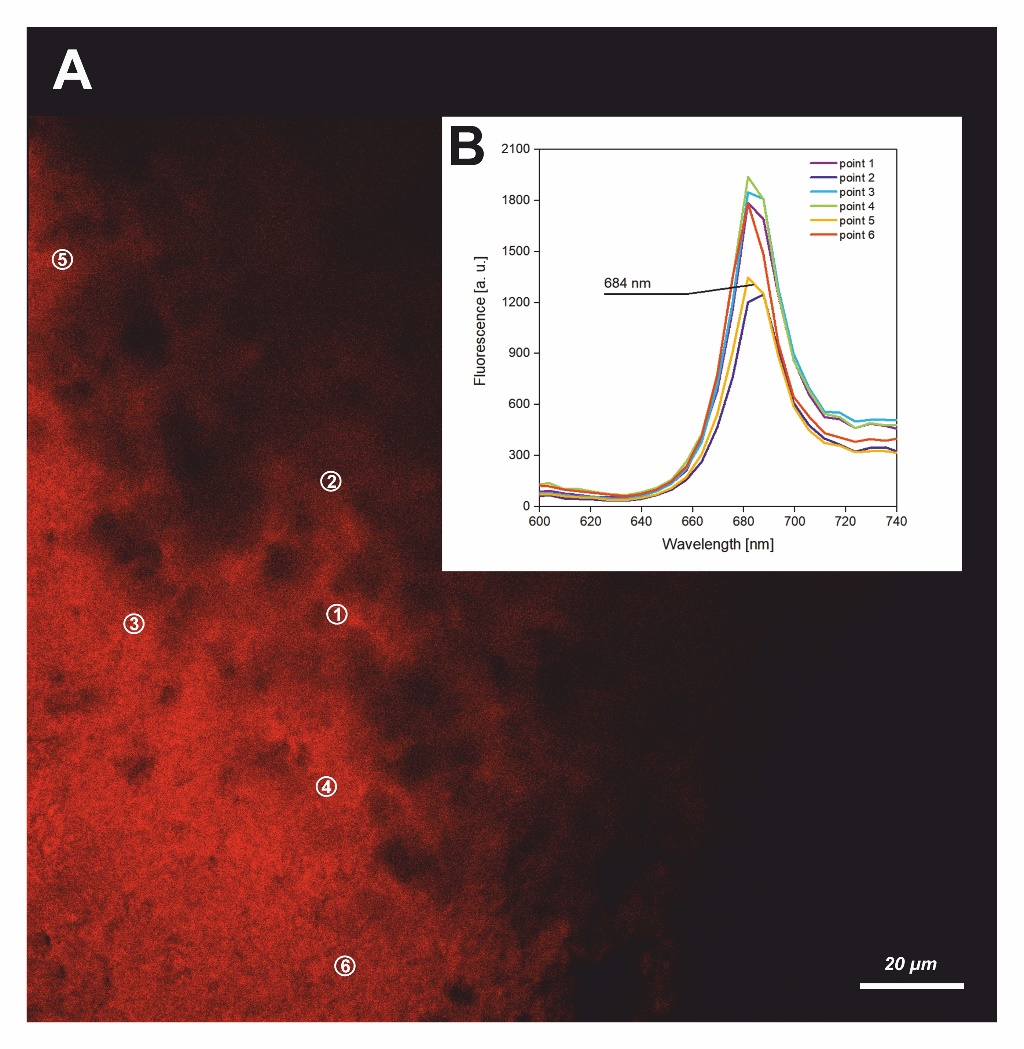


**Supplemental Figure S11**

The room temperature fluorescence emission spectra of LHCII_3_ recorded by confocal laser scanning microscopy (CLSM) in the GE-LHCII_3_-PPBQ system after 20 min of OCP treatment.

The image presents the overall Chl fluorescence volume at the boundary of the graphite working electrode (**A**). The circles with numbers indicate the points where the non-normalized fluorescence spectra were registered (**B**).

The CLSM image was made with a Nikon A1R MP inverted microscope equipped with a 32-channel resonant scanner and a Nikon S Plan Fluor ELWD 40x Ph2 ADM lens. The fluorescence excitation was applied with a 561 nm diode laser and the fluorescence emission was detected in the range from 585 to 745 nm with the 6 nm spectral resolution. The image was obtained under *in situ* experiment with the use of a special holder (Schematic diagram S2). Because graphite is not visible in CLSM, the red fluorescence corresponds to the position of LHCII_3_ inside GE. The data obtained are in agreement with our previous investigations ^3^.

**Supplemental Table S1**

Average potentials of peaks at the voltammograms registered for oxidation and reduction processes of GE-PPBQ and LHCII_3_-GE-PPBQ under dark and light conditions.

|  |  | **Square-wave voltammetry (SWV)** | | | |
| --- | --- | --- | --- | --- | --- |
| Peak number |  | I (V) | II (V) | III (V) | IV (V) |
| Experimental systems |  |  |  |  |  |
| GE-PPBQ dark |  | ­0.235 ± 0.002^a^ | 0.130 ± 0.001^a,c^ | – | −0.232 ± 0.005^a,c^ |
|  |  |  |  |  |  |
| GE-LHCII_3_-PPBQ dark |  | ­0.268 ± 0.016^a^ | 0.214 ± 0.035^a^ | – | −0.279 ± 0.032^a^ |
|  |  |  |  |  |  |
| GE-PPBQ light |  | −0.232 ± 0.004^b^ | 0.150 ± 0.010^b,c^ | 0.481 ± 0.009^b^ | −0.224 ± 0.003^b,c^ |
|  |  |  |  |  |  |
| GE-LHCII_3_-PPBQ light |  | −0.257 ± 0.015^b^ | 0.214 ± 0.025^b^ | 0.539 ± 0.016^b^ | −0.260 ± 0.021^b^ |

The average values ± SD were calculated from 4 to 7 independent experiments. Values marked with letters indicate a significant difference (p < 0.05) between GE-PPBQ and GE-LHCII_3_-PPBQ measured under dark (^a^) or light (^b^) conditions as well as between dark and light measurements for GE-PPBQ (^c^).

Differential pulse voltammetry (DPV) and square-wave voltammetry (SWV) are the pulse voltammetric methods, in which the modulation of potential is used to increase the rate and sensitivity of measurements. In these methods, the current is measured at two points for each pulse. The first measure occurs just before the pulse is applied, and the second measure takes place at the end of the pulse. The results of the subtraction of these currents are plotted as a function of potential. The DPV (Table 1) and SWW differ in pulse characterization, sensitivity, and scanning rate. Furthermore, the background currents are subtracted in SWV. Thus, some differences between the potential values can be observed.

**Data availability**

The datasets used and/or analyzed during the current study available from the corresponding author on reasonable request.

**References for Supplemental Information**

1 Mazur, R. *et al.* Specific Composition of Lipid Phases Allows Retaining an Optimal Thylakoid Membrane Fluidity in Plant Response to Low-Temperature Treatment. *Frontiers in Plant Science* **11**, 723, doi:10.3389/fpls.2020.00723 (2020).

2 Mazur, R. *et al.* Dark-chilling and subsequent photo-activation modulate expression and induce reversible association of chloroplast lipoxygenase with thylakoid membrane in runner bean (Phaseolus coccineus L.). *Plant Physiol Bioch* **122**, 102-112, doi:10.1016/j.plaphy.2017.11.015 (2018).

3 Piotrowska, P. *et al.* Electrochemical characterization of LHCII on graphite electrodes - Potential-dependent photoactivation and arrangement of complexes. *Bioelectrochemistry* **127**, 37-48, doi:10.1016/j.bioelechem.2019.01.005 (2019).

4 Kirchhoff, H., Hinz, H. J. & Rosgen, J. Aggregation and fluorescence quenching of chlorophyll a of the light-harvesting complex II from spinach in vitro. *Biochimica et Biophysica Acta* **1606**, 105-116 (2003).

5 Shukla, M. K. *et al.* A novel method produces native light-harvesting complex II aggregates from the photosynthetic membrane revealing their role in nonphotochemical quenching. *The Journal of Biological Chemistry* **295**, 17816–17826, doi:10.1074/jbc.RA120.016181 (2020).

6 Compton, R. G. & Banks, C. E. *Understanding Voltammetry, 3rd edition*. (World Scientific Publishing Europe, 2018).

7 Zhang, Y., Magdaong, N., Frank, H. A. & Rusling, J. F. Protein film voltammetry and co-factor electron transfer dynamics in spinach photosystem II core complex. *Photosynthesis Research* **120**, 153-167, doi:10.1007/s11120-013-9831-4 (2014).

8 Kuroiwa, Y., Kato, Y. & Watanabe, T. Negative shift of chlorophyll a oxidation potential by aggregation in acetonitrile/ionic liquid mixed solvents. *Journal of Photochemistry and Photobiology A: Chemistry* **202**, 191-195, doi:doi.org/10.1016/j.jphotochem.2008.12.006 (2009).

9 Kobayashi, M. *et al.* Redox potential of chlorophyll d in vitro. *Biochimica et Biophysica Acta* **1767**, 596-602, doi:10.1016/j.bbabio.2007.02.015 (2007).

10 Guin, P. S., Das, S. & Mandal, P. C. Electrochemical Reduction of Quinones in Different Media: A Review. *International Journal of Electrochemistry*, 816202, doi:<https://doi.org/10.4061/2011/816202> (2011).

11 Karsili, T. N. V., Tuna, D., Ehrmaier, J. & Domcke, W. Photoinduced water splitting via benzoquinone and semiquinone sensitisation. *Physical Chemistry Chemical Physics* **17**, 32183, doi:10.1039/c5cp03831f (2015).

12 Huynh, M. T., Colin, W. A., Cavell, A. C., Stahl, S. S. & Hammes-Schiffer, S. Quinone 1 e– and 2 e–/2 H+ Reduction Potentials: Identification and Analysis of Deviations from Systematic Scaling Relationships. *Journal of American Chemical Society* **138**, 15903-15910, doi:10.1021/jacs.6b05797 (2016).

13 Lingvay, M., Akhtar, P., Sebők-Nagy, K., Páli, T. & Lambrev, P. H. Photobleaching of Chlorophyll in Light-Harvesting Complex II Increases in Lipid Environment. *Frontiers in Plant Science* **11**, 849, doi:10.3389/fpls.2020.00849 (2020).

14 Lambrev, P. H. *et al.* Functional domain size in aggregates of light-harvesting complex II and thylakoid membranes. *Biochimica et Biophysica Acta* **1807**, 1022-1031, doi:10.1016/j.bbabio.2011.05.003 (2011).

15 Müh, F., Madjet, M. E. A. & Renger, T. Structure-Based Identification of Energy Sinks in Plant Light-Harvesting Complex II. *Journal Physical Chemistry B* **114**, 13517-13535, doi:10.1021/jp106323e (2010).

16 Lambrev, P. H., Akhtar, P. & Tan, H. S. Insights into the mechanisms and dynamics of energy transfer in plant light-harvesting complexes from two-dimensional electronic spectroscopy. *Biochimica et Biophysica Acta* **1861**, 148050, doi:10.1016/j.bbabio.2019.07.005 (2020).

17 Liu, Z. *et al.* Crystal structure of spinach major light-harvesting complex at 2.72 A resolution. *Nature* **428**, 287-292, doi:10.1038/nature02373 (2004).

18 Lyskov, S. *et al.* Serverification of molecular modeling applications: the Rosetta Online Server that Includes Everyone (ROSIE). *PloS one* **8**, e63906, doi:10.1371/journal.pone.0063906 (2013).

19 Wan, T. *et al.* Crystal structure of a multilayer packed major light-harvesting complex: implications for grana stacking in higher plants. *Molecular Plant* **7**, 916-919, doi:10.1093/mp/ssu005 (2014).

20 Combs, S. A. *et al.* Small-molecule ligand docking into comparative models with Rosetta. *Nature Protocols* **8**, 1277-1298, doi:10.1038/nprot.2013.074 (2013).

21 Schaller, S. *et al.* The main thylakoid membrane lipid monogalactosyldiacylglycerol (MGDG) promotes the de-epoxidation of violaxanthin associated with the light-harvesting complex of photosystem II (LHCII). *Biochimica et Biophysica Acta* **1797**, 414-424, doi:10.1016/j.bbabio.2009.12.011 (2010).

22 Randviir, E. P. & Banks, C. E. Electrochemical impedance spectroscopy: an overview of bioanalytical applications. *Analytical Methods* **5**, 1098-1115, doi:10.1039/c3ay26476a (2013).
